# Antibody-functionalized lipid nanocarriers for RNA-based cancer gene therapy: advances and challenges in targeted delivery

**DOI:** 10.1039/d5na00323g

**Published:** 2025-08-22

**Authors:** Nadine Wafik Nabih, Hatem A. F. M. Hassan, Eduard Preis, Jens Schaefer, Asaad Babker, Anass M. Abbas, Muhammad Umair Amin, Udo Bakowsky, Sherif Ashraf Fahmy

**Affiliations:** a Organic and Medicinal Chemistry Department, Faculty of Pharmacy, University of Sadat City Sadat City Menoufia 32897 Egypt; b Medway School of Pharmacy, University of Kent Chatham Maritime Kent ME4 4TB UK; c Department of Pharmacy, Institute of Pharmaceutics and Biopharmaceutics, Marburg University Robert-Koch-Str. 4 35037 Marburg Germany ubakowsky@aol.com sherif.fahmy@pharmazie.uni-marburg.de sheriffahmy@aucegypt.edu; d Department of Medical Laboratory Sciences, College of Health Sciences, Gulf Medical University Ajman United Arab Emirates; e Department of Clinical Laboratory Sciences, College of Applied Medical Sciences, Jouf University Sakaka 72388 Saudi Arabia

## Abstract

Despite remarkable advances in cancer therapeutics, conventional treatments still face significant hurdles, including systemic toxicity, poor tumor specificity, multidrug resistance, and suboptimal intracellular delivery. Lipid-based nanocarriers (LBNCs) have emerged as versatile platforms for delivering therapeutic RNA molecules, offering biocompatibility and tunable properties that enhance drug stability and bioavailability. Functionalizing these nanocarriers with antibodies has unlocked new potential for achieving precise tumor targeting, leveraging the overexpression of specific receptors on cancer cells. This review provides a comprehensive and focused update on recent developments in antibody-decorated LBNCs designed for RNA-based cancer gene therapy. We discuss cutting-edge advances in conjugation chemistries, including site-specific strategies such as strain-promoted click reactions and Fc-glycan engineering, as well as the integration of emerging antibody formats, including nanobodies and single-domain antibodies. Furthermore, we present studies reporting the various LBNC formulations, including liposomes, solid lipid nanoparticles, lipid nanoparticles, and hybrid systems, highlighting their physicochemical characteristics, *in vitro* and *in vivo* performance, and the critical trade-offs between targeting specificity and endosomal escape efficiency. Epidemiological data underscore the pressing need for such innovations, particularly in aggressive and hard-to-treat cancers. While promising, clinical translation remains hindered by challenges in scalable manufacturing, regulatory approval, and biological complexity. Continued interdisciplinary research is essential to transform antibody-functionalized LBNCs from experimental strategies into clinically viable solutions for next-generation, RNA-based cancer therapies.

## Abstract 2

Despite remarkable advances in cancer therapeutics, conventional treatments still face significant hurdles, including systemic toxicity, poor tumor specificity, multidrug resistance, and suboptimal intracellular delivery.

## Graphical abstract

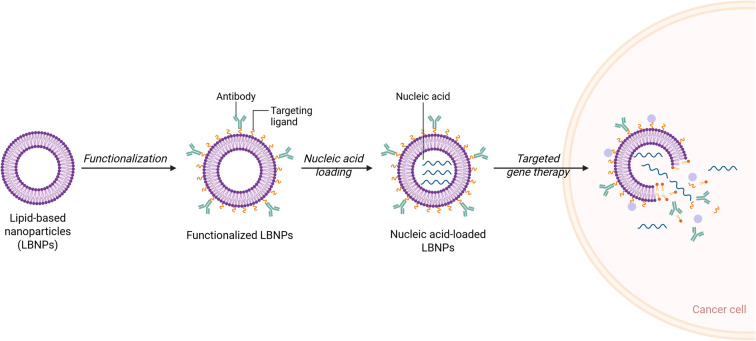

## 1. Introduction

**Fig. 1:**
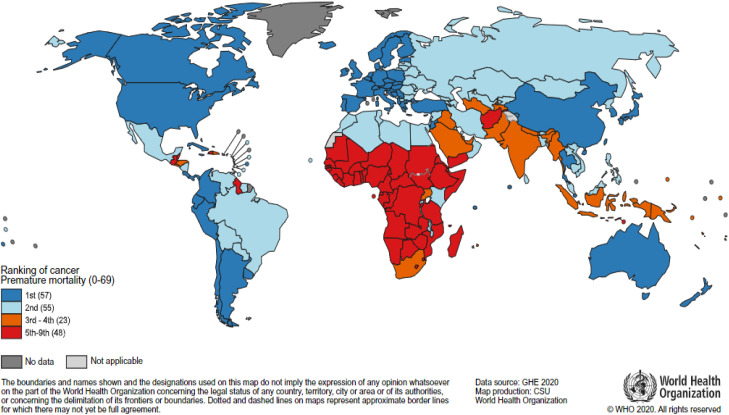
National ranking of cancer as a cause of death at ages <70 years in 2020. This figure has been adapted from ref. 1 permission from WHO, copyright (2020).

Worldwide, cancer remains one of the leading causes of death and poses a significant obstacle to increasing global life expectancy. According to estimates from the World Health Organization (WHO), cancer ranks as the first or second leading cause of mortality in 112 out of 183 nations and stands as the third or fourth leading cause in an additional 23 countries for individuals under the age of 70 (Fig. 1).^1,2^ In 2020 alone, approximately 10 million deaths and 19.3 million new cancer cases were reported worldwide, according to Global Cancer Observatory (GLOBOCAN) data, underscoring the persistent burden of this complex disease.^3^

**Fig. 2:**
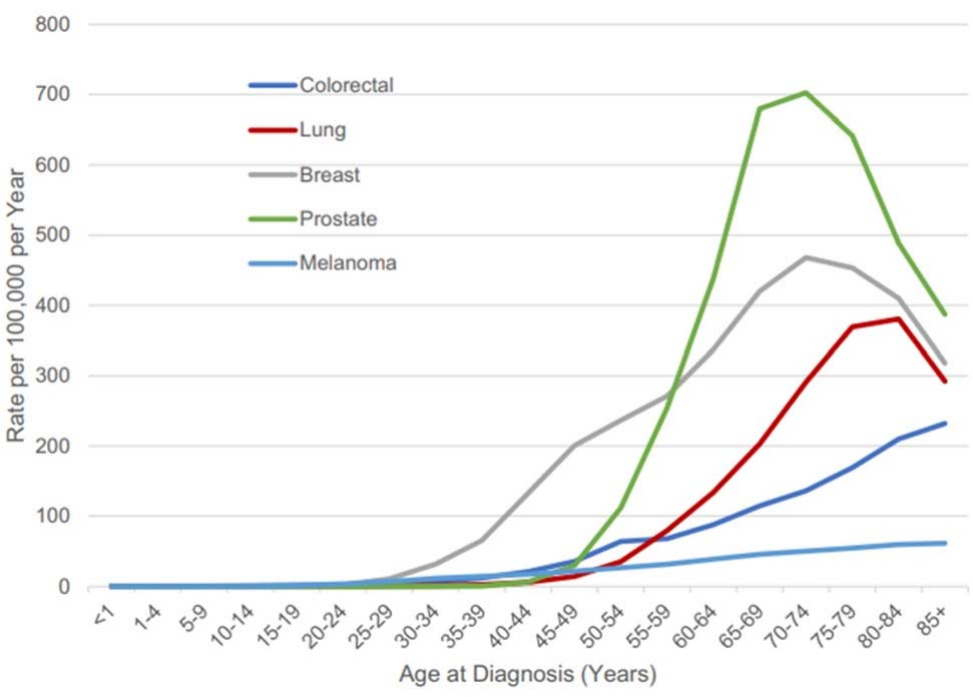
Incidence of the 5 top cancers, by age at diagnosis in the United States from 2016 to 2020. This figure has been adapted from ref. 5 permission from Oxford University Press, copyright (2024).

In the United States, recent data from the American Cancer Society (2024) indicate that five cancer types account for over half of all cancer-related fatalities: ovarian and pancreatic malignancies in women; hepatobiliary and pancreatic tumors in men; and respiratory cancers in both sexes.^4^ Moreover, cancer incidence and mortality rates rise steeply with advancing age. Between 2016 and 2020, the incidence of all cancers combined was 3.75 times higher among individuals aged 65 years and older compared to younger populations (Fig. 2).^5^

Both genetic abnormalities, accounting for roughly 10–15% of malignancies, and environmental factors play significant roles in cancer development. Environmental factors, many of which are modifiable, contribute to the remaining 85–90% of cases. These include exposure to chemical carcinogens, ionizing radiation, air pollution, viral infections, and lifestyle factors such as smoking, poor nutrition, and physical inactivity.^6,7^ In recent decades, advances in DNA sequencing and a deeper understanding of the cancer genome have transformed the landscape of oncology.^8^ Conventional cancer therapies, including radiation, chemotherapy, and other modalities, continue to form the backbone of treatment strategies.^9,10^

Radiation therapy is a commonly used therapeutic modality for solid tumors, especially after surgery.^11^ It is based on utilizing high-energy beams to kill or shrink cancer cells and halt their growth and development. While its efficacy has been proven, it is not without its challenges and harmful effects.^12^ While effective, it presents considerable challenges. Precisely targeting tumor tissues while sparing healthy structures is inherently difficult, often leading to collateral damage such as fibrosis, impaired wound healing, and cutaneous side effects ranging from erythema to blistering.^13^ Moreover, determining the optimal radiation dose required for tumor control is a challenging task and involves inherent uncertainties. Additional side effects include fatigue, alopecia, and pain.^12^

Chemotherapy, first introduced shortly after World War II, revolutionized cancer treatment, especially for metastatic disease. Today, over 200 chemotherapeutic agents are clinically available, classified into groups such as alkylating agents, antimetabolites, antibiotics, antimitotic agents, and platinum-based drugs.^14–18^ However, chemotherapy continues to face significant limitations, including a narrow therapeutic window, high rates of multidrug resistance (MDR), and systemic toxicities that damage healthy DNA and interfere with normal cell division.^19–21^ Precision medicine has increasingly steered cancer treatment toward personalized approaches, including gene-based and nucleic acid-based therapeutics. These innovative therapies hold promise for reversing disease mechanisms and addressing the shortcomings of conventional treatments.^22^ Despite their potential, numerous obstacles still limit their clinical application, such as instability in biological fluids, rapid degradation, immunogenicity, high production costs, and difficulties in achieving efficient cellular uptake and targeted tissue delivery.^23^ Consequently, substantial research has focused on developing more stable delivery systems that improve nucleic acid bioavailability and tumor targeting.^24,25^ Nanoparticles (NPs) offer a promising solution for delivering therapeutic RNAs, thanks to their capacity for passive accumulation in tumor tissues *via* the enhanced permeability and retention (EPR) effect, which arises from abnormal tumor vasculature and poor lymphatic drainage.^26–28^ Beyond passive targeting, active targeting strategies have emerged, wherein NPs are functionalized with ligands such as antibodies, peptides, aptamers, or small molecules (*e.g.*, folate) that bind selectively to overexpressed receptors on cancer cell surfaces. Ligand-receptor interactions promote receptor-mediated endocytosis, enabling nanoparticles to bypass drug efflux mechanisms and overcome multidrug resistance often encountered in cancer therapy.^29,30^

Importantly, the rationale behind recent studies focusing on antibody-functionalized lipid-based nanocarriers for RNA delivery lies in addressing several critical challenges in cancer therapy. These innovative systems aim to combine the precise tumor-targeting capabilities of antibodies with the versatile cargo-loading properties of lipid nanocarriers, offering a means to deliver therapeutic RNAs directly to cancer cells while minimizing off-target toxicity. Such platforms hold the potential to overcome biological barriers—including enzymatic degradation, immune clearance, and endosomal entrapment—that currently hinder the effective use of nucleic acid therapeutics. Moreover, the use of antibodies as targeting ligands allows for high specificity toward tumor-associated antigens, making these nanocarriers a compelling strategy for personalized and precision oncology. The studies reviewed herein were conducted to explore, validate, and optimize these strategies, seeking to translate promising preclinical findings into clinically relevant cancer treatments.

Herein, we provide a comprehensive and focused overview of recent advances in lipid-based nanoparticles, particularly those functionalized with antibodies and loaded with nucleic acids. This review highlights emerging trends and significant studies related to antibody-functionalized LBNCs engineered for active, targeted delivery of RNA therapeutics in oncology. These systems have been investigated across various cancer models to evaluate their potential for enhancing targeting precision, therapeutic efficacy, and safety while minimizing off-target effects. Collectively, these insights reflect significant paces toward next-generation, more precise, and effective cancer therapies.

## 2. Gene therapy

Gene therapy, also known as nucleic acid therapy, is a rapidly evolving field that offers novel and efficient therapeutic strategies.^22^ Nearly half a decade ago, it was conceptualized that using a genetic copy is a promising tool to overcome dysfunctional gene products produced by disrupted genes and cure both inherited and acquired disorders.^23,24^ Unlike conventional drugs that target proteins and produce a transient effect, nucleic acid therapy seeks to modulate gene expression by introducing exogenous nucleic acids. Therefore, specific, long-lasting, and curative effects are typically achieved. It is subdivided into DNA and RNA therapy. Both could employ different mechanisms to accomplish their activity, either by inhibiting or activating gene expression, adding or replacing functional genes, or editing the gene itself.^25^

Therapeutic RNAs can inhibit the expression of disease-causing genes into proteins. They are specifically designed to match specific mRNA sequences, enabling them to bind to the target mRNA and either trigger its degradation or block its translation into protein.^26^ Most recent studies have focused on the use of either mRNA, miRNA, single-guided RNA (sgRNA), or siRNA in advancing cancer therapy.

### 2.1 mRNA

**Fig. 3:**
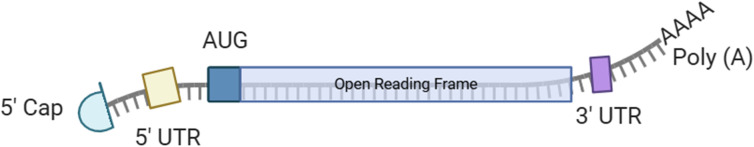
mRNA structure showing a single-stranded molecule with a 5′ cap, 5′- and 3′ UTRs surrounding the coding sequence, and a 3′ poly(A) tail. This figure has been created by BioRender https://www.biorender.com/.

mRNA therapy involves the introduction of synthetic mRNA molecules that encode functional proteins to replace missing or defective endogenous proteins in cells.^27^ mRNA molecules have a single-stranded structure with a 5′ cap, 5′- and 3′ untranslated regions (UTRs) surrounding the coding sequence, and a 3′ poly(A) tail, closely mimicking the naturally processed mature mRNA (Fig. 3).^28^ Following their administration, they are translated into the desired protein in the cytosol without entering the nucleus, rendering them suitable for various medicinal uses.^29^ Their most important application is in cancer vaccine development. mRNAs typically carry the genetic code of tumor-specific antigens (TSAs) or tumor-associated antigens (TAAs). They trigger the immunity to produce the encoded antigens and elicit an anticancer immunological response.^30^ Currently, all mRNA-based cancer vaccines administered directly are still in clinical trials.

### 2.2 miRNAs

**Fig. 4:**
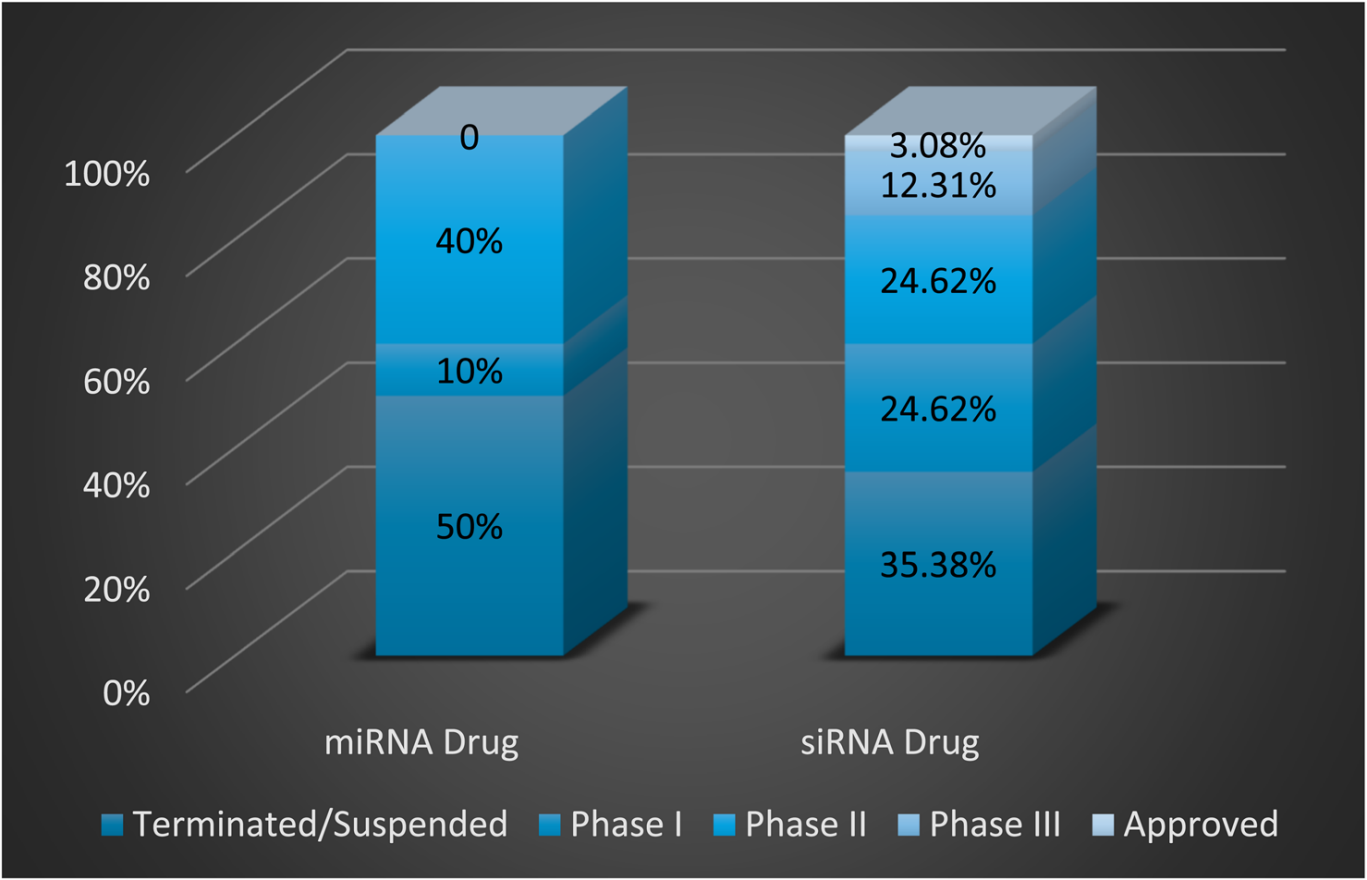
The clinical state of miRNA and siRNA drugs. 50% of miRNA drugs were suspended, 10% are in phase I, and 40% are in phase II. 35.38% of siRNA drugs were terminated, 24.62% are in phase I, 24.62% are in phase II, 12.31% are in phase III, and 3.08% are approved.

miRNAs are RNA molecules consisting of 17–25 base pairs functioning as gene regulators.^31^ It is a type of RNA interference (RNAi), a defense mechanism against exogenous genes.^32^ They bind to particular sites on mRNAs and block their translation into proteins.^33–35^ Their mechanism is very similar to that of siRNA, with slight differences. It depends on forming an RNA-induced silencing complex (RISC) through a multi-step process, which in turn identifies and binds to the target mRNA.^36,37^ miRNAs bind to the 3′ UTR of multiple target mRNAs with partial complementarity. This allows one miRNA to regulate several different genes.^38^ Compared to siRNA, no drugs have been approved by the Food and Drug Administration (FDA) to date. Approximately 50% are still in clinical trials in phases I or II, while the other 50% have been terminated, as shown in Fig. 4.^39^

### 2.3 siRNA

siRNA is a single or double-stranded, non-coding RNA molecule with between 21 and 23 base pairs. It is also a type of RNAi. It can “shut down” specific genes by targeting and breaking down their mRNA molecules.^40^ Unlike miRNA, siRNA operates through a slightly different mechanism, achieving more precise gene silencing by forming perfect Watson–Crick base pairing with target mRNA.^36,37^ Two drugs have been approved recently by the FDA. The first one was approved in 2018, ONPATTRO^®^ (patisiran, ALN TTR02) for hereditary amyloidogenic transthyretin (hATTR) amyloidosis with polyneuropathy in adults. The second one was GIVLAARI™ (givosiran, ALN-AS1) for acute hepatic porphyria (AHP).^41^

Although nucleic acids hold great potential for therapeutic and diagnostic applications, their targeted delivery into cancer cells *via* systemic administration is limited by a lack of efficient and cell-specific delivery systems. This is attributed to their susceptibility to degradation by nucleases, leading to very short half-life and unfavorable physicochemical properties that pose significant challenges, hindering effective delivery into cells to perform their therapeutic functions.^29,40,42,43^ Hence, it is essential to incorporate nucleic acids into vehicles to protect them from degradation and selectively deliver them to cancer cells.^44^

Table 1 summarizes the key points regarding RNA-based therapy.

**Table 1:** Summary of RNA-based therapy

	Structure	Mechanism	Advantages	Recent FDA-approved examples (cancer therapy)	Disadvantages
mRNA	Single-stranded RNA with 5′ cap, coding region, 5′ and 3′ UTRs, and poly(A) tail	mRNA delivers the genetic code to produce therapeutic proteins by the host's ribosomes	It can be used to produce tumor vaccines in cancer immunotherapy	All are still in clinical trials, including:- mRNA encoding neoantigen for esophageal squamous carcinoma, gastric-, pancreatic- and colorectal adenocarcinoma (NCT03468244)^45^	All RNA-based therapy suffers from: (i) Susceptibility to degradation by nucleases
				- mRNA encoding tyrosinase, glycoprotein100, melanoma-associated antigen-A3 (MAGE-A3), melanoma-associated antigen-C2 (MAGE-C2), and preferentially expressed antigen in melanoma (PRAME) and TriMix (CD40L, CD70 and a co-stimulatory molecule (caTLR4)) mRNA for melanoma (NCT03394937)^46^	(ii) Unfavorable physicochemical properties
miRNA	Endogenous RNA molecules consisting of 17–25 base pairs	The guide strand of miRNA is incorporated into a protein complex forming the RISC. The latter binds to the target mRNA with partial complementarity and inhibits its translation	It can inhibit multiple oncogenes simultaneously	All are discontinued or still in clinical trials, including:- MRG-106- miR-155 targeting leukemia and lymphoma (phase I)^47^	
				- MRX34 – miRNA mimic targeting miR-34 for liver cancer (discontinued after trials)^48^	
siRNA	Short single or double-stranded RNA (21–23 nucleotides)	The guide strand of siRNA is incorporated into a protein complex, forming the RISC. The latter binds to the target mRNA with complete complementarity and cleaves it	Highly specific gene silencing	- ONPATTRO^®^ for hATTR^49^	
				- GIVLAARI™ for AHP^50^	

## 3. Antibody-decorated lipid-based nano delivery system

**Fig. 5:**
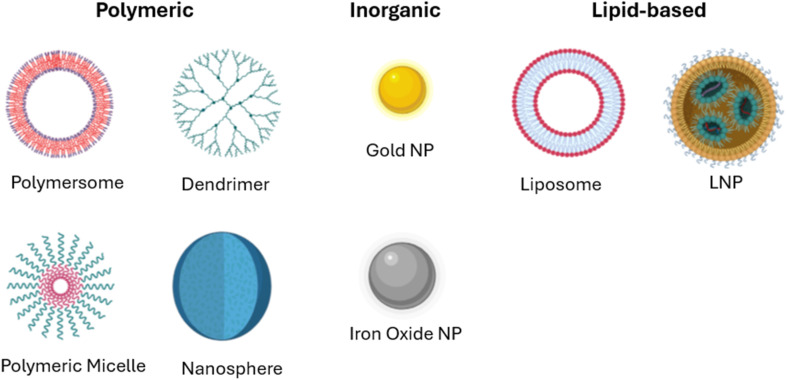
Different types of nanocarriers, including polymeric (like polymersomes, dendrimers, polymer micelles, and nanospheres), inorganic (including iron oxide NPs, and gold NPs), and LBNPs (like liposomes and lipid nanoparticles (LNPs)). This figure has been created by BioRender https://www.biorender.com/.

Nanocarriers are systems designed for targeted drug delivery, enhancing drug stability, sustaining the release of some molecules, and improving their solubility for systemic delivery. They are also adapted for therapeutic agents' protection from enzymatic degradation *via* nucleases and proteases. Due to their small nano-range size, they can extravasate into tumor tissues or penetrate microcapillaries, allowing selective target accumulation and efficient drug uptake.^51^ Several types of NPs, micelles, or liposomes are used as potential nanocarriers for different anticancer therapeutics, as illustrated in Fig. 5. However, the use of LBNCs shows great promise in the formulation of effective RNA-based cancer nanotherapeutics.

### 3.1 Lipid-based nanocarriers

Lipid-based nanocarriers are considered promising nucleic acid delivery systems in clinical settings. Compared to different nanocarriers, they possess several advantages that mark them as potential nanocarriers for different genetic materials, including adaptability, higher payload capacity, biocompatibility, and low toxicity. There are several types of LBNCs including liposomes, lipid nanoparticles (LNPs), solid lipid nanoparticles (SLNs), and nanostructured lipid carriers (NLCs).^52^ Liposomes are among the most established nanocarriers, mainly composed of cholesterol and phospholipids. They belong to the nanovesicles category.^53^ They have a bilayer structure of anionic and neutral phospholipids surrounding an aqueous core, allowing for the encapsulation of both hydrophobic and hydrophilic drugs.^31^ Possibly, cationic phospholipids could be added to facilitate the loading of nucleic acids. However, unmodified liposomes are quickly cleared by the reticuloendothelial system (RES) and need surface modifications to prolong their half-life.^54^

LNPs possess micelle structures mode, distinguishing them from traditional liposomes. They were initially developed for gene delivery, where the nucleic acids are entrapped within the core of LNP by cationic and/or ionizable lipids and further stabilized by a phospholipid monolayer containing a PEGylated lipid and sterol.^55^ They are characterized by efficient nucleic acid delivery, ease of synthesis, and small volume (0.5–1 micron).^56^ Yet, LNPs face limitations due to their low drug loading capacity and suboptimal biodistribution, which result in significant accumulation in the liver and spleen. This can lead to acute cumulative drug toxicity in these organs. In contrast, SLNs and NLCs are colloidal systems consisting of a hydrophobic core surrounded by an outer surfactant layer. In the case of SLNs, the core is made of a solid lipid, while in the case of NLCs, it consists of a blend of solid and liquid lipids (Table 2).^57,58^

**Table 2:** Types of LBNPs and a comparison between them

	Liposomes	LNP	SLN	NLC
	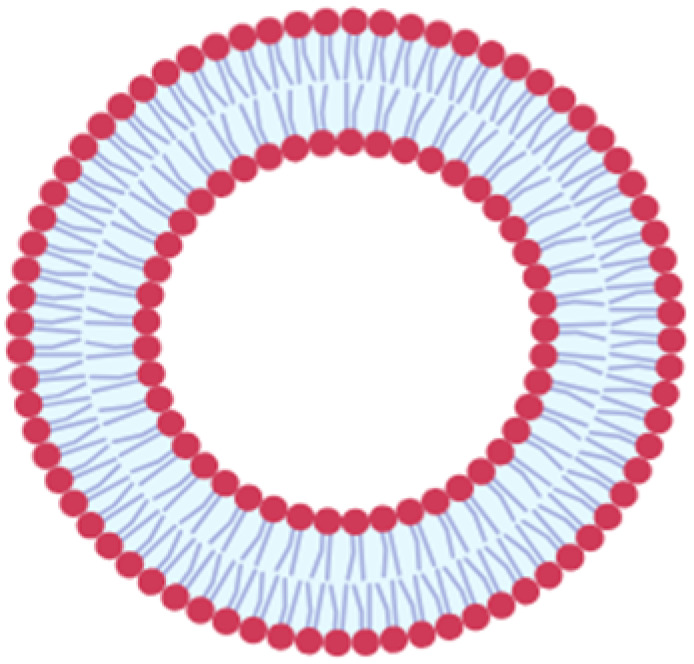	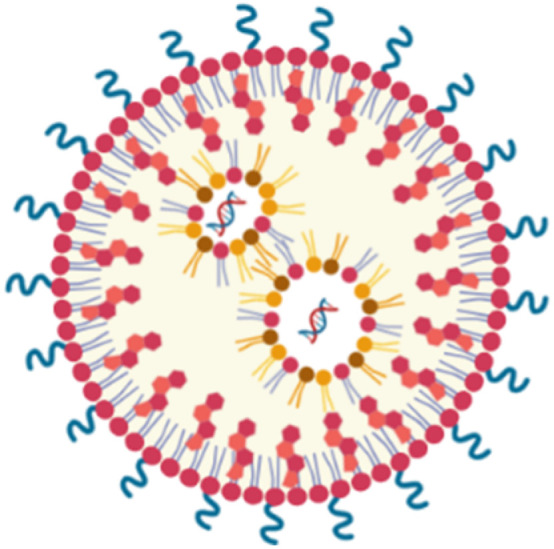	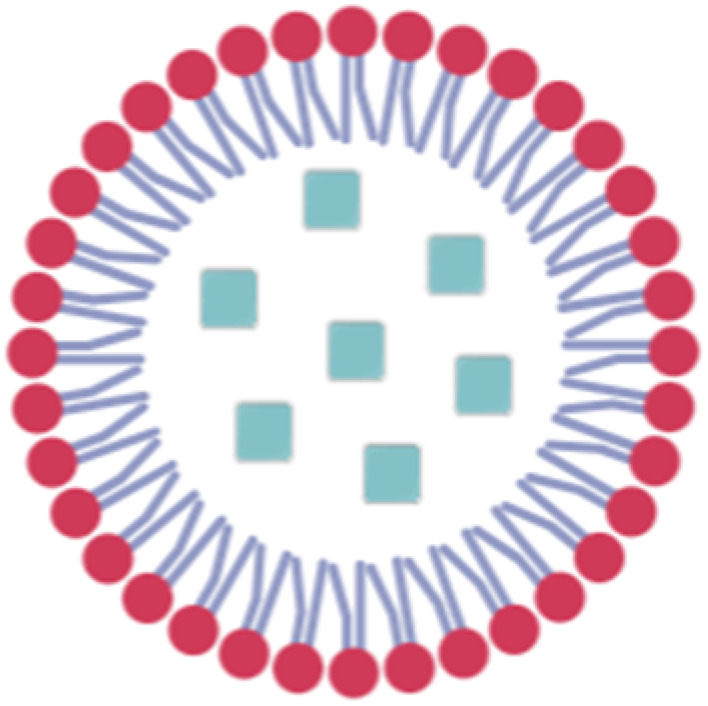	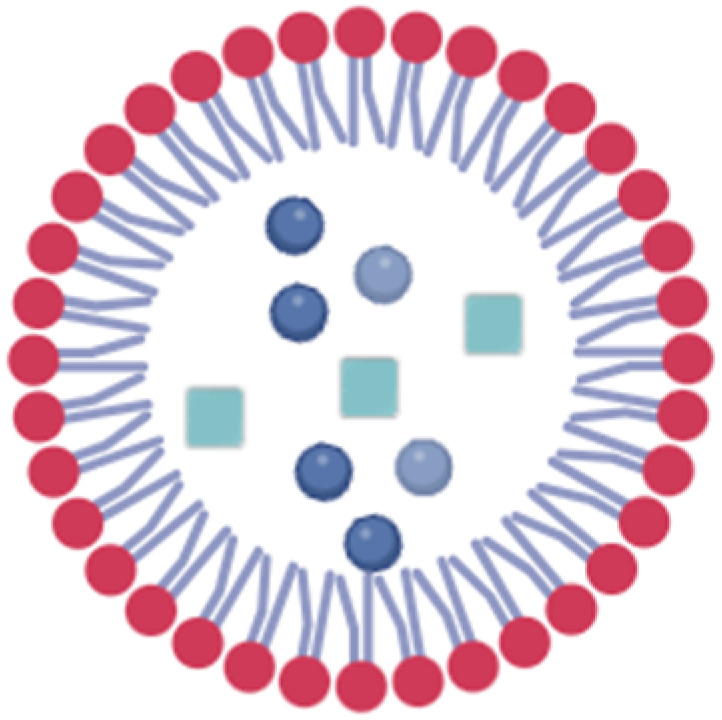
Composition	The bilayer structure of anionic and neutral phospholipids is mainly composed of cholesterol and phospholipids surrounding an aqueous internal core	It comprises cationic and/or ionizable lipids stabilized by a phospholipid monolayer containing a PEGylated lipid and sterol	Its outer layer comprises surfactants and/or co-surfactants encapsulating solid lipids. The latter is characterized by a low melting point and solidness at ambient and body temperature, offering enhanced protection of the entrapped drug compared to liposomes	Its outer layer comprises surfactants and/or co-surfactants encapsulating both solid and liquid lipids
Cargo	Hydrophobic or hydrophilic small molecules, especially siRNA and oligonucleotides	Oligonucleotides, mainly nucleic acids	Hydrophobic or hydrophilic small molecules, especially siRNA and oligonucleotides	Hydrophobic or hydrophilic small molecules
Advantages	Convenient to deliver drugs with different characteristics and have a high loading capacity	Effective nucleic acid delivery and easily synthesized	High stability and a long duration of drug release	Solid and liquid lipids lead to a less-ordered, imperfect structure, ensuring better stability and reducing the tendency to leak the drug prematurely during storage
Disadvantages	Low stability	Limited drug load, high uptake in the liver and spleen	Limited drug loading capacity	Expensive
References	54	55 and 56	59 and 57	57 and 58

### 3.2 Antibody-decorated LBNCs

The enhanced EPR effect facilitates the initial passive accumulation of NPs within tumor tissues. The decoration of NPs with targeting ligands can initiate active targeting, thereby increasing the selective delivery of the cargo to cancer cells and significantly improving the therapeutic index.^60^ The functionalization of LBNCs with various antibodies has emerged as a significant breakthrough in oncology during the past few years. They have garnered attention as an unconventional approach for targeted therapy, offering enhanced specificity and therapeutic efficacy. The functionalized LBNCs exhibited several advantages, including high tumor-targeted accuracy, specificity, wide adaptability, and minimized off-target effects.^61^ Several types of antibodies, including naked monoclonal antibodies (mAbs), bispecific antibodies (BsAbs), and immune checkpoint mAbs, were used to decorate LBNCs.

#### 3.2.1 Naked mAbs

They target tumor cells selectively *via* the Fab terminal based on TAAs. Non-conjugated mAbs perform their action through different mechanisms. The first mechanism is direct, in which the antibody targets the growth factor receptor, blocking its ligand binding or manipulating its activity.^62^ For example, cetuximab is an anti-epidermal growth factor receptor (EGFR) antibody that induces programmed cell death in cancer tissues by interfering with ligand binding and receptor dimerization.^63^ Indirect mechanisms of mAbs necessitate the involvement of the host's immune system components. They include complement-dependent cytotoxicity (CDC), antibody-dependent cellular phagocytosis (ADCP), and antibody-dependent cellular cytotoxicity (ADCC).^64,65^

Several mAbs have been recently approved in 2020, including Tafasitamab targeting CD19,^66^ Isatuximab targeting CD38,^67^ and Margetuximab targeting human Epidermal Growth Factor Receptor 2 (HER2).^68^ Tafasitamab and Isatuximab act through CDC, ADCC, and ADCP, while Margetuximab only acts through the ADCC and ADCP.

#### 3.2.2 BsAbs

**Fig. 6:**
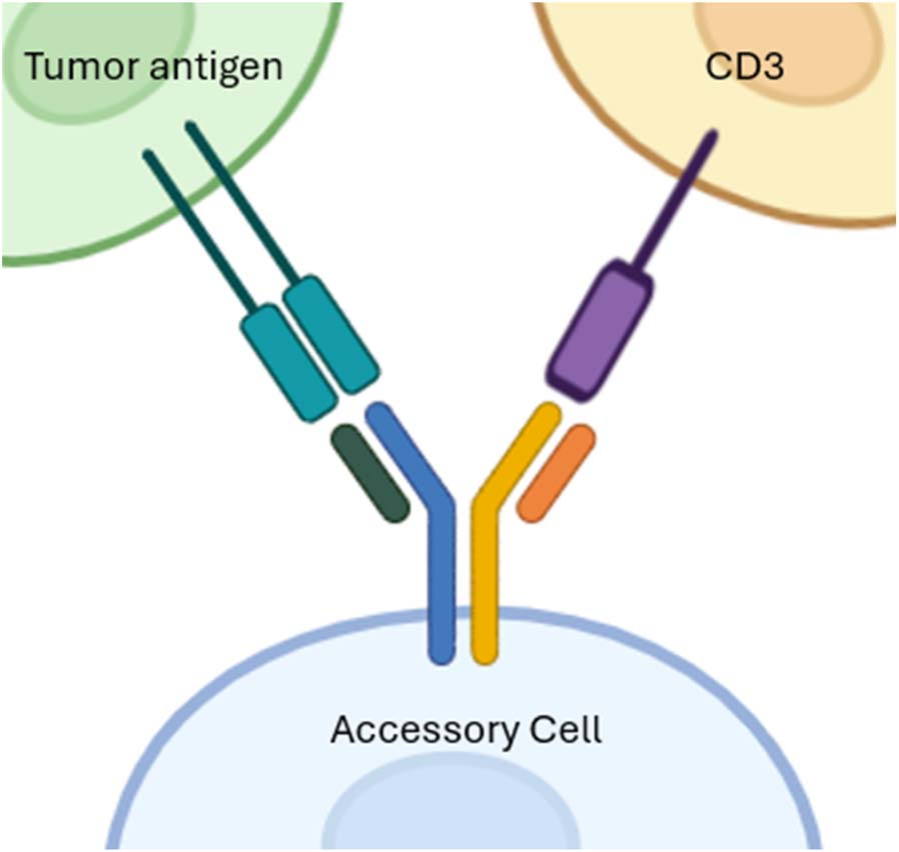
BsAb structure has an Fc region and dual functionality. BsAbs target multiple TAAs and activate cytotoxic immune T cells simultaneously while being connected to accessory cells *via* their Fc region. This figure has been created by BioRender https://www.biorender.com/.

BsAbs are produced through the conjugation of two different antibodies, which imparts dual functionality to them. They can bind simultaneously to multiple antigens, exerting a better antitumor effect.^69^ They often target multiple TAAs and concomitantly activate cytotoxic immune T cells (Fig. 6). BsAbs are classified into two types: those that bear an Fc region and those that lack it.^70^ Blinatumomab was the first FDA-approved bispecific antibody (bsAb) targeting CD19 on tumor cells and CD3^+^ cytotoxic immune T cells.^71^ In 2021, Amivantamab targeting EGFR/METR was approved.^72^

#### 3.2.3 Immune checkpoint mAbs

They target and inhibit specific immunological checkpoint proteins often involved in regulating the immune system. These proteins act as “brakes” to prevent the immune system from attacking the body's cells and are present on the surface of cancer cells and immune cells.

However, some malignancies exploit these checkpoints to evade detection and destruction by the immune system. By interfering with these inhibitory signals, checkpoint mAbs enhance the immune system's defenses against cancer cells.^73^ Several immune checkpoints have been identified, with cytotoxic T-lymphocyte-associated protein 4 (CTLA-4) and programmed cell death protein 1 (PD-1) being the most thoroughly investigated and recognized for their role in immune checkpoint blockade (ICB).^74,75^ For example, following the approval of Ipilimumab, a CTLA-4 inhibitor, for treating melanoma, patient survival rates significantly improved.^76^Table 3 summarizes the key points regarding the types of antibodies.

**Table 3:** Summary of the popularly used antibodies used to decorate LBNPs

Type	Mechanism	Advantages	FDA approved examples	Disadvantages
Naked mAbs	They target tumor cells selectively *via* Fab terminal based on TAAs, marking them for destruction by immune cells	High specificity	- Tafasitamab targeting CD19	Expensive, non-specific harmful side effects, including off-target effects and immune reaction, unknown survival rates, and prognoses
			- Isatuximab targeting CD38	
			- Margetuximab targeting HER2	
BsAbs	They can bind simultaneously to multiple antigens	Better anticancer activity through dual functionality	- Blinatumomab targeting CD19/CD3	
			- Amivantamab targeting EGFR/METR	
Immune checkpoint mAbs	Block inhibitory checkpoints like PD-1 and CTLA-4 to activate immune cells against tumors	Enhance the immune system's ability to recognize and destroy tumor cells	- Ipilimumab targeting CTLA-4 for melanoma	

Other types of antibodies include antibody fragments (such as Fab and scFv), nanobodies, and single-domain antibodies. The fragment of antigen binding (Fab) consists of an antibody light chain (VL + CL domains) connected *via* a disulfide bond to the antibody heavy chain VH and CH1 domains, and notably lacking an Fc domain. As a result, the risk of immune cell bystander activation and non-specific binding is reduced. On the other hand, they suffer from increased aggregation and low stability.^77^ Single chain fragment variable (scFv) consists of the variable regions of the light chain (VL) and heavy chain (VH) of an antibody connected by a flexible peptide linker. They have several advantages over conventional monoclonal antibodies (mAbs), including a small size that facilitates their large-scale production and tissue penetration. Yet they suffer from low thermostability and a high risk of immunogenicity.^78^ Nanobodies are one of the smallest naturally occurring antigen-binding fragments that can resist a wide pH range and high temperatures, tolerate the presence of organic solvents, are highly soluble, rarely immunogenic, and can easily penetrate tissue. However, their small size leads to rapid renal clearance and short half-life.^79^ Single-domain antibodies are considered the smallest antigen-binding units of antibodies. They are typically composed of a single variable or engineered constant domain responsible for target binding, and are commonly derived from camelids and sharks or created from human antibody domains. They are characterized by high affinity and specificity, and stability. Meanwhile, they still suffer from short half-life and limited binding surface.^80^

Beyond their structural differences, these emerging antibody formats hold particular promise for functionalizing lipid-based nanocarriers due to their unique advantages. Compared to full-length monoclonal antibodies, smaller fragments such as Fab, scFv, nanobodies, and single-domain antibodies offer reduced immunogenicity, improved tumor penetration due to their smaller size, and greater ease of large-scale manufacturing. Nanobodies, for instance, can access hidden or cryptic epitopes that full-sized antibodies cannot, potentially improving binding specificity in dense tumor microenvironments. Moreover, scFvs enable the creation of bispecific or multispecific constructs that can simultaneously target multiple tumor antigens, potentially enhancing therapeutic efficacy while mitigating off-target effects. However, despite these advantages, smaller antibody formats can have shorter circulation half-lives and may require additional modifications, such as PEGylation, to improve their pharmacokinetics. Incorporating these innovative antibody types into lipid-based nanocarriers thus represents a promising avenue for achieving precise, efficient, and safer targeted RNA delivery in cancer therapy. Future comparative studies are crucial for determining which antibody formats offer optimal performance for specific therapeutic applications.^79,80^

### 3.3 Strategies for antibody conjugation to lipid-based nanocarriers

**Fig. 7:**
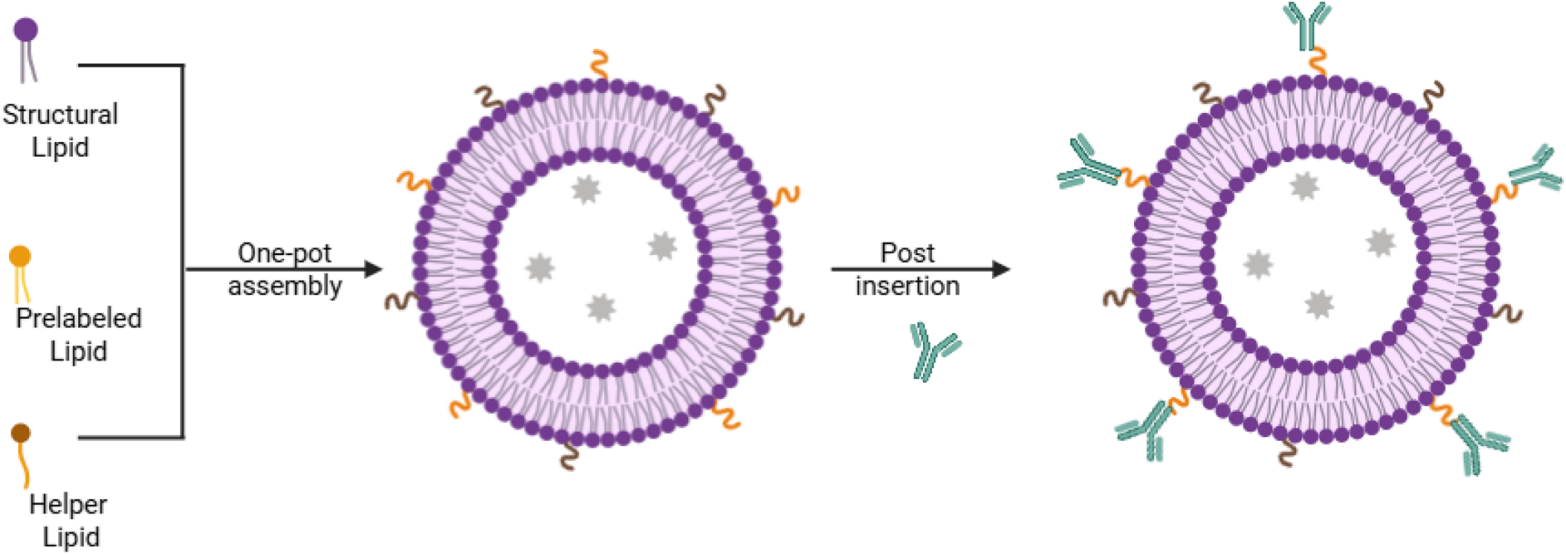
Schematic illustration of post-insertion functionalization. LBNCs are produced by combining structural lipids with PEG-lipids that have versatile terminal groups, then modifying them with antibodies. This figure was created using BioRender https://www.biorender.com/.

The one-pot assembly of lipids and targeting ligands frequently results in targeting ligands being either encapsulated within the nanoparticle core or oriented inward, making them inaccessible for receptor interaction. Consequently, post-insertion functionalization is frequently preferred to ensure that ligands remain exposed and available for effective binding. Typically, LBNCs are fabricated by mixing structural lipids with PEG-lipids featuring versatile terminal groups, such as amino, carboxyl, maleimide, or NHS functionalities, which subsequently allow chemical modification with antibodies (Fig. 7).^81^ Three broad strategies are employed to conjugate ligands—including antibodies—onto the surfaces of LBNCs:

(1) Non-specific conjugation involves techniques such as amidation, carbodiimide chemistry (EDC/NHS coupling), thiol–maleimide reactions, and click chemistry approaches, including copper-catalyzed or strain-promoted azide–alkyne cycloaddition (CuAAC or SPAAC). While these methods typically offer high conjugation efficiencies and broad ligand applicability, they often result in heterogeneous products with random ligand orientations. This misalignment can diminish the functional binding of targeting antibodies to cell-surface receptors.

(2) Site-specific conjugation was developed to address these limitations by producing homogeneous conjugates with controlled antibody orientation. Various strategies fall under this category:

• Peptide-based conjugation.

• Nickel–nitrilotriacetic acid (Ni–NTA) affinity binding for His-tagged proteins.

• Biotin–avidin binding.

• Enzymatic ligation methods, such as sortase A-mediated conjugation.

• Orthogonal click chemistries, such as strain-promoted azide–alkyne cycloaddition (SPAAC).

• Fc-glycan engineering.

Sortase A, for instance, enables site-specific antibody conjugation by covalently attaching molecules while preserving the Fab region's functionality. SPAAC reactions occur between azide groups and strained alkynes, eliminating the need for metal catalysts and providing a biocompatible solution for sensitive biomolecules. Fc-glycan engineering introduces reactive groups (*e.g.*, azides or aldehydes) into the Fc region of antibodies, enabling highly controlled conjugation through click or oxime/hydrazone chemistries and ensuring uniform Fab-out antibody orientation, which is ideal for targeted delivery. However, site-specific methods often necessitate prior modification or “coding” of targeting ligands with specific tags or terminal functionalities, adding complexity to the process.

(3) Non-covalent fusion or coating involves techniques such as the fusion of amphiphilic ligands, electrostatic-mediated adsorption, or integration of membrane protein mimics. These methods are quick and straightforward to perform but often yield conjugates with low stability and poor reproducibility under physiological conditions.^82^

Importantly, the choice of functionalization strategy significantly influences not only the physicochemical properties of the resulting LBNCs but also their biological behavior. Site-specific conjugation methods, for instance, ensure uniform antibody orientation, thereby maximizing antigen-binding accessibility and enhancing receptor-mediated endocytosis, which is crucial for the efficient intracellular delivery of therapeutic RNA. Conversely, non-specific methods may compromise targeting efficiency due to random alignment of antibodies. Moreover, emerging orthogonal chemistries and Fc-engineering approaches enable precise control over conjugation sites while preserving antibody functionality, offering avenues to optimize the delicate balance between nanoparticle stability, targeting specificity, and the ability to escape endosomal entrapment after cellular uptake. Understanding these mechanisms is crucial for designing LBNCs that can deliver RNA therapeutics effectively and safely, which remains a central challenge in the field. Notably, site-specific conjugation strategies are increasingly favored for clinical translation, as regulatory agencies emphasize the importance of homogeneous, well-characterized products with predictable biological behavior.

## 4. Studies reporting antibody-decorated LBNCs

The rationale for the growing interest in antibody-functionalized lipid-based nanocarriers for RNA delivery stems from the urgent need to overcome the critical limitations of conventional cancer treatments and earlier generations of nanoparticle systems. While traditional lipid-based carriers offer biocompatibility and efficient encapsulation of nucleic acids, they often suffer from poor tumor selectivity and rapid clearance by the mononuclear phagocyte system, reducing therapeutic efficacy and increasing systemic toxicity. By incorporating antibodies as targeting ligands, researchers aim to direct nanocarriers specifically to tumor-associated antigens, enhancing cellular uptake and minimizing off-target effects. This targeted approach not only promises higher accumulation of therapeutic RNA molecules at tumor sites but also facilitates cellular internalization through receptor-mediated endocytosis, potentially overcoming drug resistance mechanisms. Consequently, the studies summarized in this section were undertaken to investigate and optimize various strategies for antibody conjugation, nanoparticle design, and RNA payload delivery, all with the goal of developing more precise and effective gene-based cancer therapies.

In 2019, Souto *et al.*^83^ synthesized SLN linked to CAB51 antibody, an anti-human epidermal growth receptor 2 (HER2), through streptavidin–biotin interaction targeting breast cancer. SLN were synthesized by high shear homogenization and are composed of Compritol 888 ATO, cetyltrimethylammonium bromide (CTAB), and Lutrol F68. The average particle size (PS), polydispersity index (PDI), and zeta potential (ZP) were about 200 nm, 0.3, and +68 mV, respectively. At the lowest administered concentration (0.01 mg mL^−1^), the conjugated antibodies significantly enhanced the NPs' cellular uptake in HER2-overexpressing BT-474 breast cancer cells, and a synergistic effect between the SLN and CAB51 was observed. Moreover, at the same concentration, SLN-antibody conjugates decreased cell viability further to 61.0 ± 1.4% after 24 hours. However, as the concentration increased, the observed toxicity was primarily governed by the inherent toxicity of the NPs themselves. CAB51's role was primarily to enhance cellular internalization, while the plain SLN, lacking a targeting moiety, showed no significant cellular accumulation. Generally, a higher uptake rate of SLN-streptavidin-antibody complexes was observed in HER2/neu-positive BT-474 cells compared to HER2/neu-negative MCF-7 cells. This is likely attributed to its targeted interaction with the overexpressed HER2 receptors on these cells.^83^

A year later, Islek *et al.*^84^ developed dual-antibody-conjugated all-trans retinoic acid (ATRA) -loaded lipid NPs bearing both anti-programmed death-ligand 1 (PD-L1) and anti-4-1BB antibodies. It was designed as an alternative formulation and a potential opportunity for cancer therapy. Those nanocarriers were introduced to overcome PD-L1's poor targeting ability and ATRA's high toxicity and poor aqueous solubility. Sphingomyelin, tripalmitin, cholesterol, DSPE-PEG2000-Amine, and DSPE-PEG2000-Maleimide were all combined using the film hydration method to produce ATRA-loaded, maleimide-functionalized PEGylated SLNs. They were functionalized by attaching the DSPE-PEG maleimide group to the surface of the NPs. PDI values ranged from 0.222 to 0.382, PS was around 180 nm, and ZP was approximately 44 mV. ATRA release exhibited a biphasic pattern, achieving 76 ± 4.4% release within 24 hours under *in vitro* conditions. The synergy witnessed following SLN administration partly arises from the presence of a subset of tumor-infiltrating T lymphocytes that express both PD-1 and CD137 on their surface, leading to enhanced efficacy when targeting both receptors.^84^

Kim *et al.*^85^ designed liposomes targeted with two aptamers instead of antibodies for breast cancer. Aptamers are single-stranded DNA or RNA sequences that fold into specific 3D structures, allowing a high-affinity binding to target molecules through structural recognition, a process similar to an antigen–antibody reaction. The NPs were loaded with both DOX and indoleamine 2,3-dioxygenase-1 (IDO1) siRNA. Micelles were conjugated with anti-CD44 and anti-PD-L1 aptamers by thiol-maleimide chemistry and then were incorporated into liposomes, hence the name Aptm[DOX/IDO1]. Aptm[DOX/IDO1] was designed to inhibit PD-1/PD-L1 interaction between cancer cells and T-cells. Liposomes were generated by mixing cholesterol, DOTAP, DSPE-PEG2000, and DOPE. The ZP of Aptm[DOX/IDO1] was −1.31 mV, and the PS was 183 nm. When tested on MDA-MB-231 cell lines, the NPs demonstrated synergistic chemoimmunotherapy *via* a dual drug delivery system for modulation of the tumor microenvironment. They induced immunogenic cell death through DOX and reversed the IDO1-mediated immunosuppression *via* siRNA delivery. Enhanced CD8^+^ T cell infiltration into tumors, reduced tumor size, and inhibition of tumor metastasis in a breast cancer mouse model were observed. Aptamer-conjugated liposomes exhibited enhanced targeting and higher drug accumulation in tumor cells compared to normal ones. DOX fluorescence intensity in tumors was significantly higher than in normal tissues, indicating significant preferential uptake. Additionally, DOX release reached 36% at pH 6.8 and 50% at pH 5.2 after 48 hours, mimicking tumor microenvironment conditions.^85^

A critical factor in designing effective antibody-functionalized lipid nanocarriers is the choice of both antibody format and lipid nanoparticle type. Full-length monoclonal antibodies offer high specificity and prolonged circulation times, but can hinder endosomal escape due to their increased particle size and steric hindrance. Smaller formats, such as scFvs and nanobodies, offer better tumor penetration and lower immunogenicity but may suffer from a shorter systemic half-life. Similarly, liposomes offer flexibility in drug loading. However, they can be prone to leakage. In contrast, solid lipid nanoparticles (SLNs) and lipid nanoparticles (LNPs) provide greater stability but may present challenges in achieving high encapsulation efficiencies for certain types of RNA. Hybrid systems integrating polymers with lipids show promise for improving both stability and controlled release. Comparative analysis of these variables under different therapeutic contexts is crucial for optimizing nanocarrier design.

The type of antibody used for functionalization, linker, lipid composition, PS, PDI, ZP, targeted cells, and advantages of all LBNCs functionalized with antibodies mentioned above are summarized in Table 4.

**Table 4:** Summary of studies reporting antibody-decorated LBNPs

Type of antibody used for functionalization		Type of lipid-based nanocarrier	Linker	Lipid composition	Size (nm) and PDI	ZP (mV)	Target cells	Advantages	Reference
CAB51		SLN	Streptavidin–biotin linkage	Compritol 888 ATO, CTAB, and Lutrol F68	∼200	∼+68	MCF-7 and BT-474 cancer cells	CAB51's role was to enhance cellular internalization, while the plain SLN, lacking a targeting moiety, showed no significant cellular accumulation. SLN-antibody conjugates decreased BT-474 cell viability to 61.0 ± 1.4% after 24 hours at a 0.01 mg mL^−1^ concentration	83
					∼0.3				
Anti PD-L1 and anti-4-1BB antibody		ATRA-loaded SLNs	Thiol–maleimide chemistry (DSPE-PEG-MAL)	Sphingomyelin, tripalmitin, cholesterol, DSPE-PEG2000-amine, and DSPE-PEG2000-MAL	∼180	∼44	Cells expressing PD-L1 and 4-1BB (no specific cell line was mentioned)	ATRA release exhibited a biphasic pattern, achieving 76 ± 4.4% release within 24 hours under *in vitro* conditions. Synergistic effects combined with better T-cell responses were witnessed	84
					0.222 to 0.382				
siRNA IDO1	Anti-CD44 and anti-PD-L1 aptamers	Liposomes	Thiol–maleimide chemistry	Cholesterol, DOTAP, DSPE-PEG2000, and DOPE	183	−1.31	MDA-MB-231 cancer cell line	Aptamer-conjugated liposomes exhibited enhanced targeting and higher drug accumulation in tumor cells compared to normal ones. DOX fluorescence intensity in tumors was significantly higher than in normal tissues, indicating significant preferential uptake. Additionally, DOX release reached 36% at pH 6.8 and 50% at pH 5.2 after 48 hours, mimicking tumor microenvironment conditions	85
					N/A				

## 5. Studies reporting antibody-decorated LBNPs encapsulating therapeutic RNAs

LBNPs are a highly adaptable class of nanocarriers encapsulating diverse therapeutic agents like small molecules and nucleic acids. Their pharmaceutical potential has gained growing recognition in the past few decades in the industrial and research sectors. Recently, several studies have exploited the LBNPs for the successful delivery of genetic material like RNA and DNA since they offer multiple benefits, like safeguarding them from *in vivo* degradation and boosting their solubility and efficacy.^86^

For instance, LNPs carrying an anti-heparin-binding EGF-like growth factor antibody loaded with siRNA (αHB-EGF LNP-siRNA) targeting triple-negative breast cancer (TNBC) were developed in 2018. The loaded siRNA was designed to target the polo-like kinase 1 gene (PLK1). LNPs were formulated by encapsulating the cores with three lipids: DOPE, cholesterol, and DMPG, followed by the addition of DSPE-PEG for stabilization. DSPE-PEG was used to attach the αHB-EGF. PS, PDI, and ZP were detected and measured as follows: approximately 170 nm, 0.28, and 6 mV, respectively. *In vivo*, αHB-EGF LNP-siRNA exhibited significantly prolonged circulation, with plasma retention 6 to 9 times higher than unmodified LNP-siRNA. Tumor accumulation was slightly higher than that of the control and PEG LNPs. Confocal imaging revealed that αHB-EGF LNP-siRNA penetrated more deeply and dispersed more densely within tumor tissue, including regions distant from blood vessels, due to specific binding to HB-EGF expressed on MDA-MB-231 tumor cells. The assessment of the gene silencing activity of αHB-EGF LNP-siPLK1 against MDA-MB-231 showed an 80% reduction in PLK1 mRNA levels after treatment. Moreover, *in vivo* tests demonstrated that mice treated with the designed nanoparticles experienced significantly stronger suppression of tumor growth, reaching 45% compared to untreated mice.^87^

Few months later, for liver cancer management, cationic switchable LNPs encapsulating Sorafenib (SRF) and anti-miRNA27a, decorated with anti-GPC3 antibodies, were designed. Cationic switchable lipids (CSL3) were created to provide pH-responsive delivery of SRF. This was achieved by incorporating a pyridine ring into the solid lipid, which becomes protonated, leading to intramolecular hydrogen bonding, hydrocarbon chain alteration, and SRF release. Anti-miRNA 27a inhibited miRNA 27a, resulting in increased levels of FOXO1 and PPAR-γ, key regulators in tumor cell proliferation. Anti-GPC3's role was to guide NPs toward GPC3-overexpressing HepG2 liver cancer cells. CSL3 was combined with DSPC, DSPE-PEG2000, DSPE-PEG2000-MAL, and cholesterol to produce the final LNPs. Their PS was approximately 165 nm, PDI was 0.115, and ZP was 25 mV. 3-[4,5-Dimethylthiazol-2-yl]2,5-diphenyltetrazolium bromide (MTT) assay was conducted to assess HepG2 liver cancer cell viability. The IC_50_ of anti-GPC3 antibody-linked SRF/anti-miR27a-loaded lipid NPs was 0.71 μg mL^−1^, which was eight times lower than free SRF. Additionaly, 76% of SRF was released at acidic pH within 24 hours, compared to only 38% at physiological pH, supporting targeted intracellular delivery. Consequently, the combination of SRF, anti-miRNA 27a, and anti-GPC3 antibodies led to enhanced apoptosis, reduced tumor volume by nearly threefold compared to control, and significant tumor accumulation than in normal tissue.^32^

Meanwhile, another study aimed to design immunoliposomes encapsulating miRNA 130a/oxaliplatin and linked with PD-L1 mAbs as a combinatorial therapy for gastric cancer. miRNA 130a is a potential oncomir of both RAB5A and FOCL1 signaling pathways, leading to their downregulation. Thin film hydration followed by the ultrasonication technique was employed to combine DSPC, DSPE-PEG2000, cholesterol, DSPE-PEG2000-Mal, and DOTAP into liposomes. PD-L1 was linked through maleimide groups of the liposome. The average PS was 168 nm with a narrow PDI of 0.128. The surface charge was ∼21 mV. Both reduced Ki67^+^ cells and increased TUNEL^+^ cells were witnessed in immunohistochemical analysis, suggesting increased apoptosis. Regarding the liposomes release profile, 43% were released at acidic pH compared to 26% only in physiological pH. MTT assay showed a concentration-dependent inhibition pattern of the HGC27 gastric cancer cell line viability.^88^

Recently, engineering caveolae-targeted LNPs for delivery of mRNA to lungs was proved as a promising approach for lung cancer treatment. The lipid composition of the designed LNPs consisted of (6*Z*,9*Z*,28*Z*,31*Z*)-heptatriaconta-6,9,28,31-tetraen-19-yl 4-(dimethylamino)butanoate (Dlin-MC3-DMA) ionizable lipid, DSPC, cholesterol, dimyristoylglycerol-polyethylene glycol (DMG-PEG), and DSPE-PEG-MAL through a NanoAssemblr microfluidics setup. LNPs were functionalized with the targeted antibody *via* the interaction of the maleimide group on LNPs with Fab-C4 on the antibodies *via* a covalent bond. Nanocarriers with larger PS (∼160 nm) had the elasticity to target the plasmalemma vesicle-associated protein (PV1) present in the caveolae, enabling strong mRNA expression in pulmonary tissues. Their PDI was calculated to be 0.222, and ZP was equal to −5.43 mV. Since PV1 resides in the endothelial caveolae of the lung, decorating LNPs with αPV1 antibody promoted a 14-fold increase in lung accumulation. Confocal imaging confirmed the endothelial localization of αPV1 LNPs in lung tissue; meanwhile, mRNA translation in the lungs was even more remarkable^89^

sgRNA therapy is a novel RNA-based approach that utilizes the CRISPR-Cas9 pathway to facilitate gene editing in cancer and genetic disease therapies. Guided by sgRNA, the Cas9 enzyme creates precise double-strand DNA breaks, allowing targeted gene modification or inactivation. Hence, new LNPs carrying a single-guided RNA targeting the *PLK1* gene were designed to overcome the low editing efficiency and potential toxicity of existing CRISPR-Cas9 technology delivery systems. LNPs were obtained by mixing ionizable lipids (lipids 1, 6, 8, and 10 (Ref. 90)), DSPC, cholesterol, DMG-PEG, and DSPE-PEG. ASSET linker system (IgG2A; Anchored Secondary scFv Enabling Targeting) was used to tag LNP with anti-EGFR antibody. When tested against human ovarian cell line OV8, the resulting NPs showed a PDI of less than 0.2, indicating a uniform size distribution ranging from 71 to 80 nm. Upon tagging the LNP with anti-EGFR antibodies, 3-fold higher tumor accumulation compared to isotype controls and selective uptake into disseminated ovarian tumors was detected. RNA release and expression were very rapid, with therapeutic effects observed within 48 hours. PLK1 gene editing achieved an average of 82% efficacy in tumor cells and remained less than 1% in normal ones. Consequently, more efficient tumor growth inhibition and an 80% increase in patients' survival rate were achieved.^91^

In 2021, Kampel *et al.*^92^ developed siRNA targeting head and neck cancer caused by human papillomavirus (HPV), specifically the HPV E6 oncoprotein. siRNA E6 was further encapsulated in LNPs tagged with anti-EGFR antibodies. The uniform conjugation of anti-EGFR antibodies to the LNPs was achieved *via* the ASSET system. The NanoAssembler microfluidic mixing system was used to synthesize LNPs by combining DSPC, DMG-PEG, DSPE-mPEG, cholesterol, and cationic lipid 10. Their PS, PDI, and ZP were found to be ∼90 nm, ∼0.1, and ∼−5 mV, respectively. The circulation half-life of LNPs was short, with rapid clearance observed, yet confocal imaging confirmed their selective accumulation in tumor cells. Following testing against FaDu, 2A3, UMSCC-104, and UPCI: SCC090 head and neck cancer cell lines, this therapeutic combination decreased the tumor volume by 50% and restricted tumor progression. LNPs demonstrated significant RNA release and gene silencing, as evidenced by the low levels of E6 mRNA and reduced expression of E7 protein at 48 and 72 hours post-treatment. It also showed more significant induction of apoptosis *in vitro* and increased intracellular cargo delivery.^92^

A key antitumor mechanism of the immune system involves recruiting and activating CD8^+^ cytotoxic T cells. Higher levels of these infiltrating immune cells are associated with improved outcomes in solid tumors. Transfection of these cells involves introducing foreign genetic material to modify them for therapeutic purposes. In this context, Kheirolomoom *et al.*^93^ designed targeted LNPs to transfect T cells *in situ* with reporter genes, enabling the researchers to evaluate the behavior and characteristics of the transfected cells. Anti-CD3-targeted lipid NPs (aCD3-LNPs) were loaded with mRNA mCherry and tested to asses the success of gene transfection. Ionizable cationic lipid 2,2-dilinoleyl-4-(2-dimethylaminoethyl)-[1,3]-dioxolane (DLin-MC3-DMA) was combined with DSPC, DSPE-PEG2000, DSPE-PEG5000-Mal, DSPE-PEG2000-Cyanine7, and cholesterol for the synthesis of the LNPs. DSPE-PEG5000-Mal was used to bond the antibodies to the surface of the NPs. Liposomes had an average diameter of 71.2 nm, a PDI of 0.13, and a surface charge of ∼−9 mV. *In vitro*, aCD3-LNPs transfected and activated an average of 97% of Jurkat cells (T-cell leukemia cells). *In vivo*, LNPs had a blood circulation half-life of about 2 hours. They transfected 2–7% of circulating T cells and 2–4% of splenic T cells, inducing transient T-cell activation, depletion, migration, cytokine secretion, and changes in cell phenotype. In turn, the transfected T cells preferentially localized in tumors and tumor-draining lymph nodes had a rapid RNA release profile.^93^

**Fig. 8:**
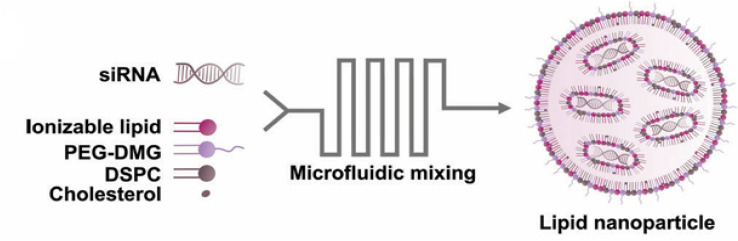
LNP preparation by mixing ionizable lipids, PEG-DMG, DSPC, and cholesterol with siRNA using microfluidic techniques. This figure has been reproduced from ref. 94 permission from Advanced Science, copyright (2023), licensed under Creative Commons Attribution 4.0 International License (https://creativecommons.org/licenses/by/4.0/). Changes: the figure was cropped for clarity.

In 2023, targeted LNPs containing a new ionizable cationic lipid were designed to target CD38, a glycoprotein overexpressed on multiple myeloma cells. They were additionally loaded with siRNA to showcase their therapeutic potential in silencing CKAP5 expression. Ionizable cationic lipids (lipid 10), PEG-DMG, DSPC, and cholesterol were mixed with siRNA using microfluidic techniques to produce the loaded LNPs (Fig. 8). Maleimide–thiol chemistry was then used to conjugate anti-CD38 antibodies to their surface. LNPs showed a 56–73 nm diameter, PDI of 0.05–0.11, and ZP ranging between −1.7 and −6.4 mV. *In vitro*, the LNPs induced cell death of 90% of multiple myeloma cells. *In vivo*, 59.4% of multiple myeloma cells in the bone marrow took up siRNA within the first 4 hours, with sustained RNA release for 24 hours. After five doses, malignant cell occupancy in bone marrow dropped from 18% to 7%, demonstrating effective targeting, rapid RNA delivery, and a strong therapeutic response.^94^

Due to the lack of hormone receptors and HER2 expression, which limits treatment options, a new approach for TNBC was explored in 2024, targeting the XBP1 gene. The XBP1 gene is known to be widely expressed in TNBC, acting as a key driver of its growth and recurrence, and is linked to reduced responsiveness. Mehta *et al.*^95^ presented a hybrid lipid polymer NP encapsulating siRNA that silences the XBP1 gene and is targeted with anti-EGFR antibodies. Poly(d,l-lactide-*co*-glycolide) acid, with a lactide: glycolide ratio of 50 : 50 (PLGA), combined with DOTAP and DSPE-PEG2000-Mal, was formulated using the double emulsion solvent evaporation technique to produce the PLGA lipid NPs. The antibodies were conjugated *via* thiol-maleimide chemistry. The PS, PDI, and ZP were approximately 230 nm, 0.3, and −3 mV, respectively. Cellular uptake in cancer cells was significantly increased, with EGFR-targeted NPs showing a 1.45-fold higher fluorescence intensity after 2 hours of incubation compared to non-targeted NPs. Besides their specificity for TNBC cells, t-PLGA NPs demonstrated higher XBP1 transfection efficiency in MDA-MB-231 cells and achieved about 75% XBP1 gene knockdown. Under hypoxic conditions, these NPs promote apoptosis and reduce XBP1 mRNA expression by approximately 90%.^95^

**Fig. 9:**
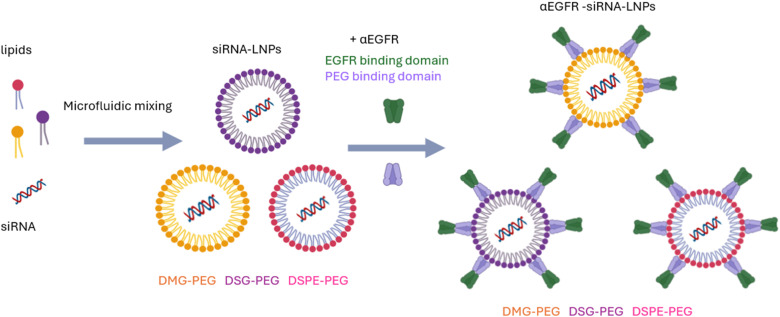
Illustration of α EGFR-siRNA-LNPs preparation. Different PEG-lipids, including DMG-PEG, DSG-PEG, and DSPE-PEG, were combined with siRNA *via* microfluidic mixing. The LNPs were then conjugated with BsAbs having dual recognition domains, one for PEG of nanocarriers and the other for neuroblastoma EGFR. This figure has been created by BioRender https://www.biorender.com/.

High-risk neuroblastoma possesses poor survival due to the ineffectiveness of conventional treatment and off-target adverse effects. In 2024, Logan *et al.*^96^ designed targeted ionizable LBNPs encapsulating pegylated siRNA PLK1 as a new treatment strategy for neuroblastoma. The attached BsAbs had dual recognition of methoxy PEG of nanocarriers and neuroblastoma EGFR. The αEGFR-siRNA-LNPs were formulated with DLin-MC3-DMA, cholesterol, DSPC, and varying PEG-lipids (DMG-PEG, distearoylglycerol-polyethylene glycol (DSG-PEG), DSPE-PEG) by microfluidic mixing process (NanoAssemblr Ignite) as shown in Fig. 9. BsAbs were non-covalently complexed with the siRNA-LNPs. Regarding the physicochemical properties, LNPs had a PS of 64.8–79.5 nm, a PDI lower than 0.3, and a ZP ranging from −16.2 to −18.7 mV. SH-SY5Y, SK-N-BE(2), and CHP-134 were used as human neuroblastoma cell lines. siRNA PLK1-LNPs achieved significant *PLK1* gene silencing by more than 2-fold and improved cell targeting by 1.2 to over 4.5 times in EGFR^+^ high-risk neuroblastoma cells. *In vivo*, tumor uptake was approximately 3 times higher than controls and persisted up to 48 hours, suggesting an acceptable circulation half-life. Among the various PEG-lipids ranging in diffusivity, formulating LNPs with DMG-PEG demonstrated the best efficacy of delivery with strong tumor selectivity, efficient siRNA delivery, and promising therapeutic potential.^96^

**Fig. 10:**
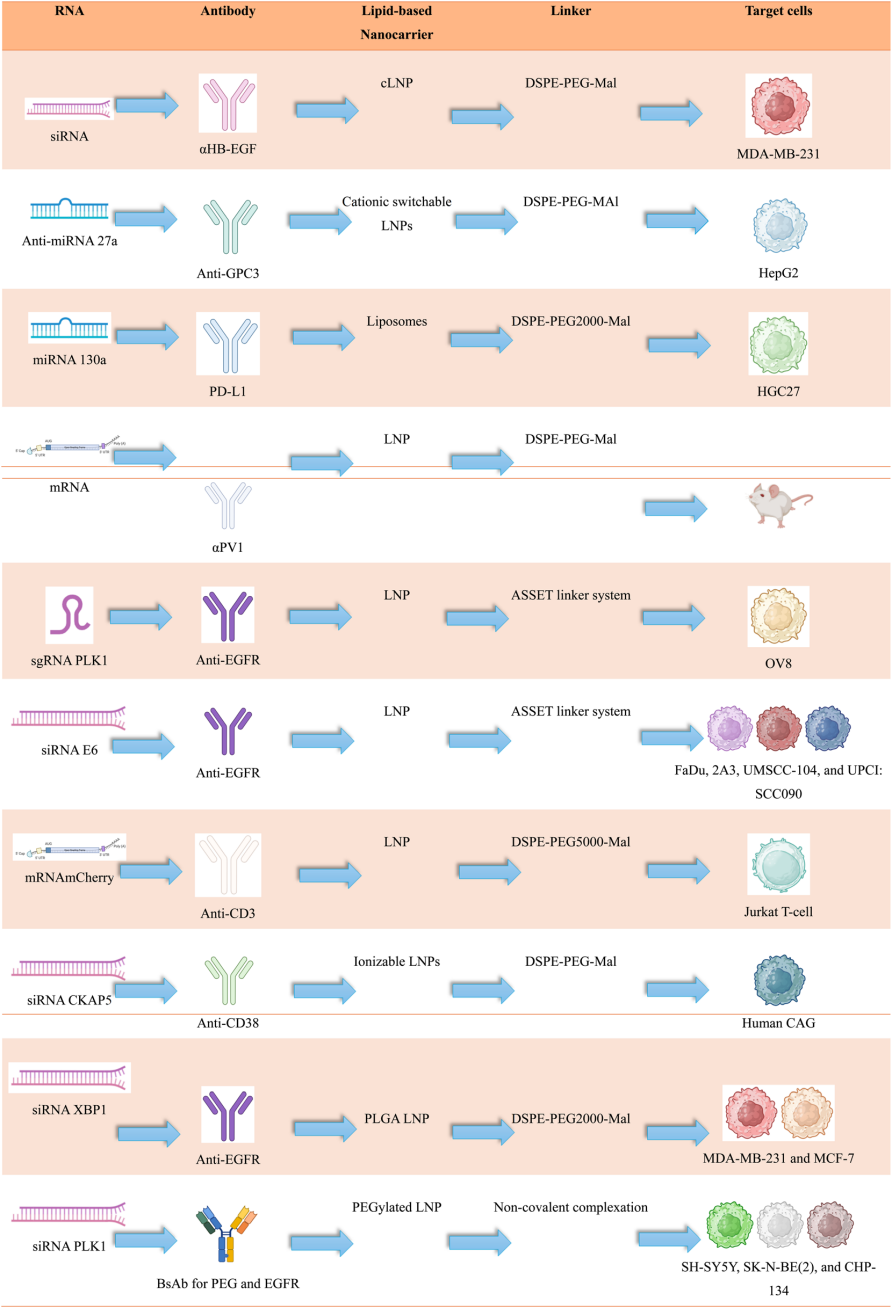
Flowchart summarizing RNA cargo types, antibody formats, lipid-based nanocarriers, conjugation linkers, and target cells discussed in this review. The schematic provides a concise visual overview complementing Table 5.

The type of loaded RNA, antibody used for functionalization, linker, lipid composition, particle size (PS), polydispersity index (PDI), zeta potential (ZP), target cells, and key advantages of all RNA-loaded antibody-functionalized lipid-based nanocarriers are summarized in Table 5. In addition, Fig. 10 provides a schematic overview grouping these elements to facilitate quick reference and complement the detailed tabular data.

**Table 5:** Summary of studies reporting antibody-decorated LBNPs encapsulating therapeutic RNAs

Type of loaded RNA	Type of antibody used for functionalization	Type of lipid-based nanocarrier	Linker	Lipid composition	Size (nm) and PDI	ZP (mV)	Target cells	Advantages	Reference
siRNA	αHB-EGF	cLNP	DSPE-PEG-Mal	DOPE, cholesterol, DMPG, and DSPE-PEG	∼170	∼6	MDA-MB-231 cancer cell line	They prolonged circulation, with plasma retention 6 to 9 times higher than unmodified LNP-siRNA. Tumor accumulation was also higher. They also penetrated more deeply within tumor tissue, due to specific binding to HB-EGF expressed on MDA-MB-231 tumor cells	87
					∼0.28				
Anti-miRNA 27a	Anti-GPC3	Cationic switchable LNPs	Thiol-maleimide chemistry (DSPE-PEG-MAL)	CSL3, DSPC, DSPE-PEG2000, DSPE-PEG2000-MAL, and cholesterol	∼165	25	HepG2 cell line	They showed an IC_50_ of 0.71 μg mL^−1^. Additionally, 76% of SRF was released at acidic pH within 24 hours, compared to only 38% at physiological pH, supporting targeted intracellular delivery	32
					0.115				
miRNA 130a	PD-L1 antibody	Liposomes	Thiol-maleimide chemistry (DSPE-PEG2000-Mal)	DSPC, DSPE-PEG2000, cholesterol, DSPE-PEG2000-Mal, and DOTAP	∼168	∼21.5	HGC27 cancer cell line	Both reduced Ki67^+^ cells and increased TUNEL^+^ cells were witnessed in immunohistochemical analysis, suggesting increased apoptosis. Regarding the liposomes' release profile, 43% were released at acidic pH compared to 26% only at physiological pH. MTT assay showed a concentration-dependent inhibition of the HGC27 viability	88
					0.128				
mRNA	αPV1 antibody	LNP	Thiol-maleimide chemistry (DSPE-PEG-Mal)	Dlin-MC3-DMA ionizable lipid, DSPC, cholesterol, DMG-PEG, and DSPE-PEG-Mal	∼160	−5.43	Tested *in vivo* only on female Balb-c (BALB/cAnNHsd) mice	Decorating LNPs with αPV1 antibody promoted a 14-fold increase in lung accumulation. Confocal imaging confirmed the endothelial localization of αPV1 LNPs in lung tissue; meanwhile, mRNA translation in the lungs was even more remarkable	89
					0.222				
sgRNA PLK1	Anti-EGFR antibody	LNP	ASSET linker system	Ionizable lipids (lipids 1, 6, 8, and 10, DSPC, cholesterol, DMG-PEG, and DSPE-PEG)	71–80	N/A	OV8 cell line	Upon tagging the LNP with anti-EGFR antibodies, 3-fold higher tumor accumulation compared to isotype controls and selective uptake into disseminated ovarian tumors was detected. RNA release and expression were very rapid, with therapeutic effects observed within 48 hours. PLK1 gene editing achieved an average of 82% efficacy in tumor cells and remained less than 1% in normal ones. Consequently, more efficient tumor growth inhibition and an 80% increase in patients' survival rate were achieved	91
					0.2				
siRNA E6	Anti-EGFR antibody	LNP	ASSET linker system (recombinant protein linker)	DSPC, DMG-PEG, (DSPE-mPEG), cholesterol, and cationic lipid 10	∼90	∼−5	FaDu, 2A3, UMSCC-104, and UPCI: SCC090 cell lines	LNPs decreased tumor volume by 50% and restricted tumor progression. They demonstrated significant RNA release and gene silencing, as evidenced by the low levels of E6 mRNA and reduced expression of E7 protein at 48 and 72 hours post-treatment	92
					∼0.1				
mRNAmCherry	Anti-CD3 antibody	LNP	Thiol-maleimide chemistry (DSPE-PEG5000-Mal)	Cationic lipid DLin-MC3-DMA, DSPC, DSPE-PEG2000, DSPE-PEG5000-Mal, DSPE-PEG2000-Cyanine7, and cholesterol	71.2	∼−9	Jurkat T-cell leukemia cells	LNPs had a blood circulation half-life of about 2 hours. The transfected T cells were preferentially localized in tumors and tumor-draining lymph nodes, with a rapid RNA release profile	93
					0.13				
siRNA CKAP5	Anti-CD38 antibody	Ionizable LNPs	Thiol-maleimide chemistry	Ionizable cationic lipids (lipid 10), PEG-DMG, DSPC, and cholesterol	56–73	−1.7 to −6.4	Human CAG cell line	*In vitro*, the LNPs induced cell death of 90% of multiple myeloma cells. *In vivo*, 59.4% of multiple myeloma cells in the bone marrow took up siRNA within the first 4 hours, with sustained RNA release for 24 hours	94
					0.05–0.11				
siRNA XBP1	Anti-EGFR antibody	PLGA LNP	Thiol-maleimide chemistry (DSPE-PEG2000-Mal)	DOTAP and DSPE-PEG2000-Mal	∼230	∼−3	MDA-MB-231 and MCF-7 cell lines	The NPs significantly enhanced cellular uptake in TNBC cells, showing a 1.45-fold increase in fluorescence after 2 hours compared to non-targeted NPs. They achieved 75% XBP1 gene knockdown and enhanced apoptosis	95
					∼0.3				
siRNA PLK1	BsAb for PEG and EGFR	PEGylated LNP	Non-covalent complexation with PEGylated LNPs	DLin-MC3-DMA, cholesterol, DSPC, and varying PEG-lipids (DMG-PEG, DSG-PEG, DSPE-PEG)	64.8–79.5	−16.2 to −18.7	SH-SY5Y, SK-N-BE(2), and CHP-134 human neuroblastoma cell lines	They achieved significant *PLK1* gene silencing by more than 2-fold and improved cell targeting by 1.2 to over 4.5 times. *In vivo*, tumor uptake was average 3 times higher than controls and persisted up to 48 hours, suggesting an acceptable circulation half-life	96
					<0.3				

## 6. Stimuli-responsive antibody-functionalized LBNCs for RNA delivery

Stimuli-responsive systems integrated into antibody-functionalized lipid-based nanocarriers (LBNCs) represent a promising strategy for achieving controlled and selective drug release in cancer therapy. These innovative nanocarriers can respond to diverse endogenous and exogenous stimuli, allowing precise activation at tumor sites while maintaining stability during circulation. Endogenous triggers include conditions specific to the tumor microenvironment, such as low pH, high redox potential, overexpressed enzymes, or unique biomarker concentrations. Exogenous stimuli, such as light, ultrasound (US), magnetic fields, radiation, and temperature changes, offer external control for spatial and temporal activation of drug release. By combining antibody-mediated targeting with stimuli-responsiveness, LBNCs can enhance tumor selectivity, improve intracellular delivery of therapeutic RNAs, and reduce off-target effects. The following section summarizes recent studies employing these strategies, detailing their design, physicochemical characteristics, and therapeutic outcomes. For instance, in 2019, Tang *et al.*^97^ designed multifunctional nanoplatforms as delivery systems for combined and targeted cancer therapy of TNBC. A carrier system for photoabsorber loading, targeted therapy for enhanced accumulation, and combination with another therapeutic modality (strategies commonly employed in photothermal treatment) have been applied in the designed NPs. They were prepared as lipid-coated calcium phosphate NPs (LCP NPs) functionalized with anti-EGFR BsAb for enhanced MDA MB-468 breast cancer cell targeting and better tumor cell accumulation. LCP NPs were loaded with cell death siRNA and indocyanine green (ICG). LBNPSs were synthesized by combining DOPA, DOPC, cholesterol, DSPE-PEG2000 Folate, DSPE-PEG2000, and DSPE-mPEG2000. The methoxy group on mPEG present on the NP surface was conjugated in a non-covalent way with the Fc region of the BsAb. PS and PDI were calculated to be 40–50 nm and 0.12–0.17, respectively. ZP ranged between −15 and −11 mV. ICG produced higher thermal energy in tumor cells following laser radiation than the free one, and both ICG and cell death siRNA induced apoptosis synergistically. Following their administration, LCP NPs demonstrated prolonged circulation and enhanced tumor accumulation, reaching 1.75% of the injected dose. Cellular uptake in MDA-MB-468 cells was significantly enhanced by up to threefold, and the siRNA release profile was pH-responsive, with around 50% released at pH 5.5 within 1 hour, and 90% by 10 hours. During *in vivo* studies in mouse models, they eliminated both small tumors (∼100 mm^3^) and large tumors (∼500 mm^3^) and raised the tumor temperature to 48.2 °C, confirming enhanced photothermal conversion.^97,98^

**Fig. 11:**
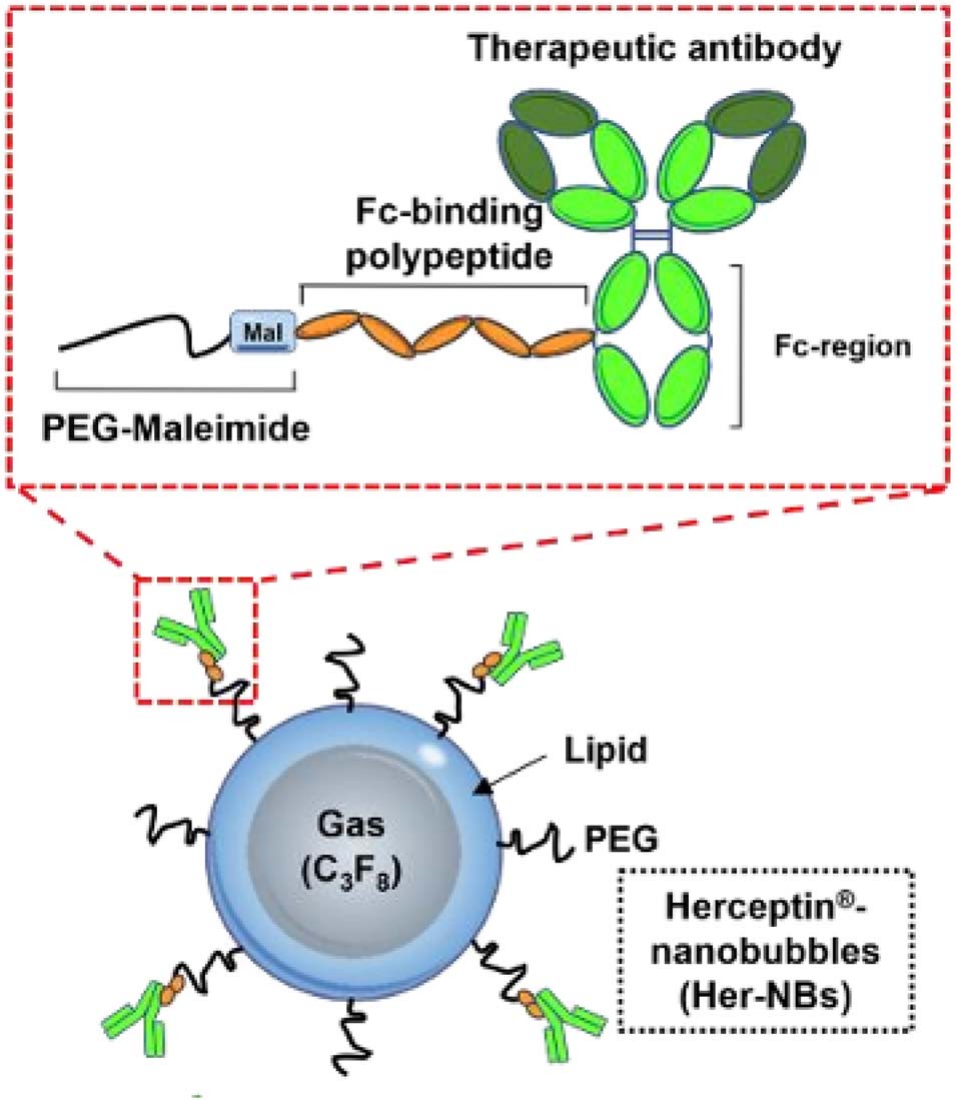
Schematic illustration of the structure of antibody-functionalized NBs. They are composed of a lipid layer bearing a PEG moiety. The Fc-G67-binding polypeptide is connected by the PEG-maleimide molecule attached to the NB surface and from the other side to the antibody's Fc-region. This figure has been reproduced from ref. 65 permission from MDPI, copyright (2023).

In 2023, Yano *et al.*^65^ developed nanobubbles (NBs), gas-filled bubbles less than 1 μm in size, loaded with Trastuzumab, demonstrating the ability to effectively infiltrate solid tumor microenvironments and deliver targeted antibodies into breast cancer cells. The NBs were synthesized from DSPC, DSPE-PEG2000, and DSPE-PEG2000-Mal by the reverse-phase evaporation vesicle method. The polyethylene glycol (PEG) on the lipid surface of the NBs was coupled with an Fc-G67 binding polypeptide that, in turn, was easily attached to the antibody's Fc region (Fig. 11). NBs showed high homogeneity; their PS and ZP were about 172 nm and −22 mV, respectively. These polypeptides exhibit strong binding affinities due to their origin from bacterial proteins (protein A/G), which enables efficient and quick antibody loading. Upon administration, NBs were subjected to therapeutic ultrasound (TUS). They remained visible in tumor vasculature on US imaging for up to 5 minutes post-injection, indicating short circulation time. This is because TUS triggers the expansion of NBs and their subsequent collapse, resulting in the perforation of blood vessels and promoting the accumulation of antibodies within the tumor tissues. Moreover, targeted uptake was confirmed by fluorescence imaging, which showed strong binding to HER2-positive SKOV3 ovarian cancer cells and minimal binding to HER2-negative MDA-MB-231 cells.^65^

**Fig. 12:**
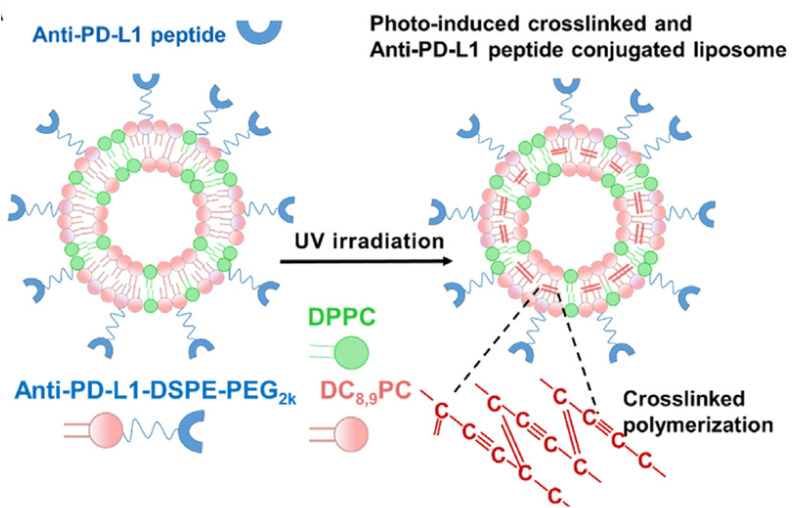
Schematic illustration of ICB-liposome preparation. Both DPPC and DC_8,9_PC form the liposome lipid bilayer. The anti-PD-L1 is conjugated to the lipid surface *via* DSPE-PEG2000. Upon exposure to UV, DC_8,9_PC undergoes cross-linked polymerization, enhancing liposome stability and delaying its degradation. This figure has been reproduced from ref. 73 permission from Elsevier, copyright (2024).

Unlike other studies, in 2024, Lee *et al.*^73^ proposed photo-induced cross-linked and anti-PD-L1 peptide-incorporated ICB-liposomes as a potential ICB cancer therapy. ICB-liposomes are developed using a formulation consisting of the phospholipid 1,2-bis(10,12 tricosadiynoyl)-*sn*-glycero-3-phosphocholine (DC_8,9_PC), DPPC, and an anti-PD-L1 peptide-conjugated DSPE-PEG2000. DC_8,9_PC facilitates intermolecular crosslinking within the liposomal bilayer when exposed to ultraviolet (UV) light, enhancing the stability and functionality of the liposomes (Fig. 12). Liposomes were prepared by the film casting method, giving rise to LBNPs with low PDI, PS of around 138 nm, and ZP of about ∼−3 mV. A robust cytotoxic T lymphocyte-mediated antitumor immunity was triggered, and PD-L1 degradation was sustained for 72 hours, unlike anti-PD-L1 monoclonal antibodies (mAbs), which exhibited rapid PD-L1 recycling within 9 hours. *In vivo*, the circulation half-life of ICB-liposomes + UV was extended to 18.3 ± 2.1 hours, longer than PEG-liposomes (4 hours) and ICB-liposomes-UV (6 hours). In CT26 murine cancer cells, tumor accumulation was also higher, exhibiting up to 2.1-fold greater fluorescence intensity in tumor tissues, indicating enhanced passive and active targeting. Antitumor efficacy was marked by a reduction in tumor volume, reaching 463 ± 145 mm^3^ on day 13, and extended survival for more than 20 days.^73^

The type of loaded RNA and Ab used for functionalization, type of stimulus, linker, lipid composition, PS, PDI, ZP, targeted cells, and advantages of all RNA-loaded lipid NP functionalized with antibodies mentioned above are summarized in Table 6.

**Table 6:** Summary of studies on stimuli-responsive antibody-functionalized lipid-based nanocarriers for RNA delivery

RNA cargo type	Antibody type	Nanocarrier type	Stimulus	Linker	Lipid composition	Size (nm)/PDI	ZP (mV)	Target cells	Advantages	Ref.
siRNA	Anti-EGFR BsAb	LCP NPs	Light	Methoxy group of PEG (mPEG)	DSPE-mPEG2000, DSPE-PEG2000 folate, DSPE-PEG2000, DOPA, DOPC, and cholesterol	40–50	−15 to −11	MDA-MB-468 cell line	LCP NPs demonstrated prolonged circulation and enhanced tumor accumulation, achieving a concentration of 1.75% of the injected dose. Cellular uptake in MDA-MB-468 cells was significantly enhanced by up to threefold, and the siRNA release profile was pH-responsive, with around 50% released at pH 5.5 within 1 hour, and 90% by 10 hours. They also eliminated small and large tumors	97
						0.12–0.17				
—	Trastuzumab	NBs	TUS	Fc-G67 binding polypeptide	DSPC, DSPE-PEG2000, and DSPE-PEG2000-Mal	∼172	∼−22	SKOV3 and MDA-MB-231 cancer cells	NBs remained visible in tumor vasculature on US imaging for up to 5 minutes post-injection, indicating short circulation time. Fluorescence imaging demonstrated strong binding to HER2-positive SKOV3 ovarian cancer cells and minimal binding to HER2-negative MDA-MB-231 cells, confirming targeted uptake	65
						Narrow size distribution				
—	Anti-PD-L1	Liposomes	UV	DSPE-PEG2000	DPPC and DC_8,9_PC	∼138	∼−3	CT26 cancer cells	Their circulation half-life was extended to 18.3 ± 2.1 hours. Tumor accumulation was also substantially higher, with 2.1-fold greater fluorescence intensity in tumor tissues. Antitumor efficacy was marked by reduced tumor volume and extended survival (>20 days)	73
						Low PDI				

Beyond the individual studies summarized above, stimulus-responsive systems integrated into antibody-functionalized LBNCs represent a highly promising strategy for achieving precise, controlled release of RNA therapeutics directly at tumor sites. These systems exploit the unique characteristics of the tumor microenvironment, such as acidic pH, high levels of reducing agents like glutathione, overexpressed enzymes, or externally applied triggers like light and ultrasound, to trigger nanoparticle destabilization or cargo release. For example, pH-sensitive lipids facilitate endosomal escape *via* the proton sponge effect, while redox-sensitive linkers cleave in response to intracellular thiols, ensuring cytosolic release. Light and ultrasound can provide spatial and temporal precision, enabling activation only at desired tumor sites. Importantly, integrating stimuli-responsive features into antibody-decorated LBNCs can help resolve the critical trade-off between high targeting specificity and efficient intracellular delivery, as these systems remain stable during systemic circulation but activate selectively within tumor tissues. However, future research should focus on ensuring the safety, scalability, and regulatory feasibility of these complex multifunctional systems to translate them into clinical applications.

## 7. Clinical trials

Although antibody-functionalized LBNCs for RNA delivery represent an exciting area of research, their translation into clinical practice remains in its early stages. To date, no antibody-decorated lipid-based nanocarriers (LBNCs) or antibody-functionalized RNA-loaded LBNCs have advanced into clinical trials or preclinical cancer therapy pipelines. However, the rapid clinical progress of RNA-loaded lipid nanocarriers in oncology provides a strong technological foundation for potential future applications involving antibody-targeting. Notably, anti-Transferrin Receptor (anti-TfR) scFv-modified liposomes carrying plasmid DNA, such as SGT-53 and SGT-94, have been evaluated in clinical studies, demonstrating the feasibility of antibody-modified lipid nanocarriers for nucleic acid delivery.^99,100^ While these examples involve DNA rather than RNA payloads, they serve as important precedents for targeted nucleic acid therapies using antibody-functionalized lipid systems. Several innovative RNA-based LBNCs have entered clinical trials for cancer treatment, albeit without antibody modifications. BioNTech, for example, has developed multiple RNA-based immunotherapies. In the NCT02410733 trial, an off-the-shelf RNA-lipoplex vaccine targeting shared melanoma antigens elicited robust CD4^+^ and CD8^+^ T-cell responses, particularly when combined with anti-PD-1 therapy.^101^ Additional trials, including NCT03289962, NCT03815058, and NCT04486378, have explored personalized RNA-lipoplexes tailored to each patient's tumor-specific mutations. These personalized vaccines demonstrated significant immunogenicity, achieving strong T-cell responses in 73% of patients and notable disease stabilization.^102^ Meanwhile, Moderna has maintained an active oncology pipeline focusing on RNA-based therapeutics delivered *via* lipid nanocarriers. In the NCT03739931 trial, the mRNA-2752 formulation was administered intratumorally in patients with high-risk ductal carcinoma *in situ*, yielding high response rates and strong local immune activation.^103^ Although these systems currently do not employ antibody functionalization, their clinical success highlights the feasibility and safety of lipid-based RNA delivery, which could be further enhanced through the incorporation of targeting antibodies in future developments. Overall, while antibody-decorated LBNCs for RNA-based cancer therapy have not yet entered the clinical stage, the accelerating progress in RNA-LBNC platforms from companies such as Moderna, BioNTech, and Arcturus underscores the growing potential of these technologies. It is anticipated that ongoing research and technological advancements will soon bridge this gap, paving the way for clinical translation of antibody-functionalized RNA nanomedicines.

## 8. Clinical translation of antibody-decorated LBNC

The translation of antibody-decorated lipid-based nanocarriers (LBNCs) into clinical practice holds significant promise for targeted cancer gene therapy. However, it faces numerous challenges, including those related to manufacturing, regulatory pathways, safety considerations, and economic feasibility.

### 8.1 Manufacturing and scalability

Manufacturing antibody-decorated LBNCs at scale requires precise control over nanoparticle composition, size distribution, antibody conjugation efficiency, and batch-to-batch reproducibility. Production must comply with Good Manufacturing Practice (GMP) guidelines, ensuring robust quality control, sterile processing, and validated analytical methods to characterize critical quality attributes (CQAs). Challenges include maintaining consistent antibody orientation and density on the nanoparticle surface, preventing aggregation, and ensuring the stability of both lipid carriers and RNA payloads during manufacturing and storage. Emerging continuous manufacturing techniques, microfluidic systems, and automated high-throughput processes are being investigated to enhance scalability and reduce production costs for complex nanomedicines.

### 8.2 Regulatory and safety considerations

Regulatory approval pathways for antibody-decorated LBNCs remain complex and evolving. Nanoparticles incorporating biologics, such as antibodies, are classified as combination products in many jurisdictions, requiring a comprehensive evaluation of both the drug and device components. Regulatory agencies, such as the U.S. Food and Drug Administration (FDA) and the European Medicines Agency (EMA), increasingly demand detailed characterization of nanoparticle physicochemical properties, biodistribution, immunogenicity, and potential toxicity. One particular challenge is the lack of universally accepted preclinical models that reliably predict how these complex nanocarriers will behave in humans. Immunogenic responses to both the lipid components and surface-bound antibodies may complicate safety assessments and necessitate tailored regulatory guidance. Collaboration among regulatory bodies, industry stakeholders, and academic researchers is crucial for developing harmonized guidelines and expediting the approval processes for nanomedicines.

### 8.3 Cost-effectiveness and market viability

Another key barrier to clinical translation is the economic feasibility of antibody-decorated LBNCs. While these systems offer superior targeting and therapeutic precision compared to naked LNPs or simpler peptide-functionalized nanoparticles, their increased manufacturing complexity and stringent quality requirements significantly raise production costs. Cost-effectiveness analyses are crucial to evaluate whether the clinical benefits of antibody-functionalization justify the added expenses, especially in oncology settings where multiple targeted therapies may already be available. Compared to simpler naked LNP systems, which have achieved rapid regulatory approval for mRNA vaccines, these present a formidable benchmark for manufacturing costs and scalability. However, antibody-decorated systems may prove cost-effective in highly personalized medicine scenarios where precise tumor targeting yields significant therapeutic benefits and reduced off-target toxicities.

Despite these challenges, substantial progress is being made toward the clinical translation of antibody-decorated LBNCs. Advances in antibody engineering, scalable manufacturing technologies, and regulatory science are gradually reducing barriers. Ongoing clinical trials and preclinical pipelines from companies such as Moderna, BioNTech, and Arcturus, though currently focused on non-antibody LNPs, provide a valuable framework for future translation of targeted LBNC systems. Multidisciplinary collaboration among researchers, clinicians, regulatory agencies, and industry will be critical for transforming the promise of antibody-decorated LBNCs into clinical reality.^104^

## 9. Conclusions and future directions: balancing targeting specificity and endosomal escape in antibody-functionalized lipid nanocarriers

Achieving precise tumor targeting without off-target accumulation remains one of the most significant challenges in nanomedicine. While many nanoplatforms for drug delivery rely on passive targeting through the enhanced permeability and retention (EPR) effect, this mechanism often suffers from randomness and a lack of control, underscoring the need to develop more effective active targeting strategies. Beyond antibodies, ligands like aptamers can form precise three-dimensional structures with high affinity for specific cell surface markers. However, their low molecular weight can lead to rapid renal clearance. Similarly, small chemical ligands, such as folic acid, have been employed to exploit overexpressed tumor-specific receptors, but they may lack the binding specificity and multivalency offered by antibody-based systems.

In addition to targeting specificity, intracellular bioavailability represents a crucial and often rate-limiting factor in the success of gene therapy. Nanocarriers typically enter cells *via* endocytosis, becoming trapped in endosomal or lysosomal compartments, where their cargo may be degraded, thus reducing therapeutic efficacy. Various strategies have been developed to promote endosomal escape, including membrane fusion, pore formation, and incorporating pH-sensitive moieties (*e.g.*, hydrazine or imidazole groups) that induce the proton sponge effect. Attaching endosome-disrupting peptides or polymers to nanocarriers has also shown promise in overcoming this intracellular barrier.

A central theme highlighted throughout this review is the inherent trade-off between enhancing targeting specificity and maintaining efficient endosomal escape. Decorating lipid-based nanocarriers with bulky targeting ligands such as full-length antibodies can substantially improve tumor selectivity. However, it may simultaneously hinder endosomal disruption due to increased particle size or altered surface charge. Thus, while increased targeting specificity can improve tumor accumulation and therapeutic index, it may come at the cost of reduced intracellular release efficiency. Balancing these competing priorities remains a fundamental challenge in nanocarrier design. Emerging strategies, such as employing smaller antibody fragments (*e.g.*, scFvs, nanobodies) and adopting precise site-specific conjugation techniques, hold promise for preserving high targeting specificity while minimizing steric and charge-related barriers to endosomal escape. These approaches may help maintain optimal nanoparticle size and surface characteristics that are crucial for intracellular delivery. This trade-off also has important implications for clinical translation, as larger, more complex nanocarriers can pose manufacturing and regulatory challenges, emphasizing the need for innovative design strategies that balance complexity with scalability and safety.

Future research must systematically investigate how ligand density, orientation, and nanoparticle physicochemical properties influence both tumor accumulation and endosomal escape efficiency. Ultimately, overcoming the inherent trade-offs between targeting specificity and intracellular delivery efficiency will be pivotal for translating antibody-functionalized LBNCs from promising laboratory constructs into clinically effective cancer therapeutics.

Substantial progress has been made in recent years toward developing antibody-functionalized lipid-based nanocarriers (LBNCs) for RNA-based cancer gene therapy. Advances in modern conjugation techniques, such as strain-promoted azide–alkyne cycloaddition (SPAAC), enzymatic ligation with sortase A, and Fc-glycan engineering, now enable more precise and site-specific attachment of antibodies, preserving their orientation and biological activity. Furthermore, emerging antibody formats, including nanobodies, single-chain variable fragments (scFvs), and bispecific antibodies, offer smaller size, reduced immunogenicity, and enhanced tumor penetration, presenting exciting opportunities for future therapeutic designs. Despite these encouraging developments, significant barriers remain on the path to clinical translation. Manufacturing and scalability challenges must be addressed to ensure the reproducibility and cost-effectiveness of antibody-decorated lipid nanoparticles. Regulatory considerations, particularly those related to safety and immunogenicity, necessitate comprehensive evaluation through rigorous preclinical and clinical studies. Cost-effectiveness analyses will be essential to determine whether the complexity and expense of antibody-targeted nanocarriers justify their benefits over simpler systems, such as naked LNPs or peptide-based targeting approaches. Moreover, comparative analyses across different nanocarrier platforms, antibody formats, and RNA cargos are crucial for guiding rational design choices. To date, few studies have quantitatively compared parameters such as targeting efficiency, circulation half-life, tumor accumulation, or therapeutic efficacy between various systems. Future research should aim to generate head-to-head data that can inform which strategies are optimal under specific clinical circumstances.

While numerous studies report promising results for antibody-functionalized lipid-based nanocarriers, direct quantitative comparisons among different formulations remain limited. Future research should prioritize head-to-head studies that systematically evaluate critical parameters such as targeting efficiency, systemic circulation half-life, tumor accumulation ratios, endosomal escape efficiency, and overall therapeutic efficacy across various nanocarrier types and antibody formats. Such comparative analyses are crucial for guiding rational design choices and identifying optimal delivery strategies for specific clinical contexts. Notably, reported tumor accumulation efficiencies for antibody-decorated LNPs vary widely, ranging from approximately 5% to over 25% of the injected dose, depending on antibody density and nanoparticle composition. In this review, we have summarized all available quantitative metrics reported in the literature, including circulation half-life, tumor accumulation *versus* normal tissue accumulation, intracellular uptake percentages, and RNA release profiles, consolidating them into the Advantages column of Table 6 and throughout the discussion sections. However, broader comparative studies across platforms remain essential to establish clear performance benchmarks for future clinical applications.

From a future perspective, ongoing advances in nucleic acid biology, lipid chemistry, and nanocarrier engineering will continue to drive innovation in this field. The integration of stimuli-responsive elements, such as pH- or redox-sensitive components, offers additional avenues for improving site-specific release and overcoming intracellular barriers. The increasing availability of clinical data from companies such as Moderna, BioNTech, and Arcturus further underscores the real-world potential of lipid-based RNA delivery systems, including those incorporating antibody-based targeting.

In conclusion, antibody-functionalized LBNCs represent a highly promising approach to achieving precise, efficient, and safe delivery of RNA-based therapeutics for cancer treatment. While significant challenges remain, interdisciplinary research efforts focusing on molecular design, mechanistic understanding, and translational development are poised to transform these technologies into clinically viable solutions that could reshape the future of cancer therapy.

## Conflicts of interest

There are no conflicts to declare.

## Data Availability

This manuscript does not involve any experimental work.
